# Structural and functional analysis of the *Acinetobacter baumannii* BlsA photoreceptor and regulatory protein

**DOI:** 10.1371/journal.pone.0220918

**Published:** 2019-08-15

**Authors:** Cecily R. Wood, Mariah S. Squire, Natosha L. Finley, Richard C. Page, Luis A. Actis

**Affiliations:** 1 Department of Microbiology, Miami University, Oxford, Ohio, United States of America; 2 Department of Chemistry and Biochemistry, Miami University, Oxford, Ohio, United States of America; Consejo Superior de Investigaciones Cientificas, SPAIN

## Abstract

The *Acinetobacter baumannii* BlsA photoreceptor has an N-terminal (NT) BLUF domain and a C-terminal (CT) amino acid sequence with no significant homology to characterized bacterial proteins. In this study, we tested the biological role of specific residues located in these BlsA regions. Site-directed mutagenesis, surface motility assays at 24°C and protein overexpression showed that residues Y7, Q51 and W92 are essential for not only light-regulated motility, but also BlsA’s solubility when overexpressed in a heterologous host. In contrast, residues A29 and F32, the latter representing a difference when compared with other BLUF-containing photoreceptors, do not play a major role in BlsA’s biological functions. Analysis of the CT region showed that the deletion of the last five BlsA residues has no significant effect on the protein’s light-sensing and motility regulatory functions, but the deletion of the last 14 residues as well as K144E and K145E substitutions significantly alter light-regulated motility responses. In contrast to the NT mutants, these CT derivatives were overexpressed and purified to homogeneity to demonstrate that although these mutations do not significantly affect flavin binding and photocycling, they do affect BlsA’s photodynamic properties. Notably, these mutations map within a potential fifth α-helical component that could play a role in predicted interactions between regulatory partners and BlsA, which could function as a monomer according to gel filtration data. All these observations indicate that although BlsA shares common structural and functional properties with unrelated photoreceptors, it also exhibits unique features that make it a distinct BLUF photoreceptor.

## Introduction

*Acinetobacter baumannii* is a Gram-negative opportunistic pathogen and the causative agent of both nosocomial- and community-acquired infections [1]. Although *A*. *baumannii* is commonly associated with hospital infections, it has been isolated from a wide range of environmental sources and samples including water and aquaculture environments [2], soil [3], varied food sources [4], animals [5, 6] and insects [7], all of which could be reservoirs for this bacterium outside the hospital environment [8]. *A*. *baumannii’*s prevalence in the environment and hospital settings and its ability to spread throughout the community can be attributed to its capacity to sense and respond accordingly to different environmental cues, including its ability to sense light through the blue-light-sensing protein A (BlsA) [9]. BlsA is a blue light sensing using FAD (BLUF) domain photoreceptor protein involved in controlling biofilm, motility and virulence functions in response to light at temperatures lower than 30°C [9, 10]. BlsA shares a similar architecture with other BLUF photoreceptors: the flavin chromophore is inserted between the first two N-terminal (NT) α helices of the BLUF domain, which also includes a 5-standed β-sheet component [11]. BlsA’s NT BLUF domain, which is predicted to noncovalently bind the flavin chromophore through conserved and semiconserved amino acids including a tyrosine-glutamine pair and a tryptophan residue, is hypothesized to act as a light sensor for this microorganism. The light signal, which is generated by proton-coupled electron transfer (PCET), is initiated by the conserved tyrosine upon blue light illumination [12] and then transmitted to the C-terminus (CT) of BlsA through rearrangements of an intricate hydrogen-bonding network involving the side chains of the conserved glutamine and other semiconserved amino acids of the BLUF domain around the N5 of the isoalloxazine ring of the flavin chromophore. Hydrogen bond switching between the glutamine side chain, the flavin and other residues in the BLUF domain are proposed to trigger a signal through the strand β_5_ of the protein, particularly through the side chain of the tryptophan residue, which leads to conformational changes in the BlsA structure and, ultimately, potential complex formation with unknown target proteins [13]. UV-visible spectroscopic studies can reveal the signaling state of BLUF domains characterized by a 10-nm redshift, which is indicative of photoexcitation [14]. The light state is reversible and, depending on the BLUF protein, relaxation back to the dark-adapted state occurs in as little as 10 seconds (t_1/2_, 5 s), as in the case of PixD (Slr1694), or can take up to 30 minutes (t_1/2_, 15 min), as with the large BLUF protein AppA [15, 16]. Previous studies have demonstrated that BlsA is intermediate between these proteins and relaxes back to its dark state after illumination with a half-life of approximately 8 minutes [13].

Although the basic photochemistry and architecture of the BLUF photoreceptors are similar, the proteins are further classified into one of two groups: “long” multidomain BLUF-containing proteins or “short” BLUF proteins. Unlike the first and one of the best-characterized BLUF domain-containing photoreceptors, AppA, which is a multidomain BLUF blue light sensing protein [17], BlsA is considered a short BLUF-containing photoreceptor protein. Like other short BLUF proteins, such as Tll0078 [18], the first 96 amino acids of BlsA are predicted to be dedicated to light sensing, whereas the remaining 60 CT residues have no known function. Because their CT regions show no sequence similarity to known DNA-binding motifs or other proteins involved in regulation of gene expression, these short photoreceptor proteins are predicted instead to participate in protein-protein interactions with downstream effectors involved in regulatory functions as previously proposed [17].

Structural and photochemical characterizations of the BLUF domains of different long and short bacterial photoreceptors have been the focus of several studies since the identification of this family of light sensing proteins. However, the biological role of the divergent CT regions, particularly those associated with short light sensing proteins that do not display predictable output signal domains, as it pertains to the organism remains to be elucidated.

In this work we focused on the biological function and biochemical properties of BlsA by targeting specific amino acid residues of the N- and C-terminal regions. This approach showed that amino acid residues located in the NT region predicted to interact with the flavin adenine dinucleotide (FAD) chromophore are critical not only for BlsA’s light-dependent regulatory functions, but also for protein stability when overexpressed in a heterologous host. The analysis of BlsA’s CT region showed that although the last five amino acid residues are dispensable for its photosensing and regulatory roles, the 7-residue region located between amino acids 144 and 150 could represent an unidentified functionally significant fifth α helix component. Surface motility assays showed that deletion or site-directed mutagenesis of residues mapped immediately preceding or within the fifth α-helix region affect the ability of isogenic derivatives to display differential motility responses at 24°C upon illumination. Additionally, size exclusion chromatography resulted only in the isolation of BlsA monomers without any indication of dimerization or oligomerization of this protein under the native conditions used in this work. Taken together, our results provide critical insights that contribute to understanding the structure-function relationships of a photosensory and regulatory protein that plays a key role in *A*. *baumannii*’s pathophysiology.

## Materials and methods

### Bacterial strains, culture conditions and plasmids

All strains and plasmids used in this study are listed in Table 1. *Escherichia coli* and *A*. *baumannii* strains were routinely cultured in Luria Bertani (LB) broth or on agar plates and supplemented with appropriate antibiotics when necessary [19]. Swimming broth (SB; 10 g/l tryptone, 5 g/l NaCl) and swimming agar (SA; SB containing 0.3% agarose) were used for liquid cultures and motility assays, respectively. *A*. *baumannii* was routinely grown at 37°C for 16–18 h (overnight).

**Table 1:** Bacterial strains and plasmids used in this work.

Strains/plasmids	Relevant characteristic(s)^a^	Source/reference
Strains		
*A*. *baumannii*		
ATCC 17978 (17978)	Wildtype clinical isolate	ATCC
OR	*blsA*::*aph* derivative of 17978; Km^R^	[9]
OR.V	OR harboring empty pMU368; Km^R^; Zeo^R^	This work
OR.W	OR harboring pMU1202; Km^R^; Zeo^R^	This work
OR.Y7A	OR harboring pMU1229; Km^R^; Zeo^R^	This work
OR.A29S	OR harboring pMU1222; Km^R^; Zeo^R^	This work
OR.F32N	OR harboring pMU1213; Km^R^; Zeo^R^	This work
OR.Q51A	OR harboring pMU1226; Km^R^; Zeo^R^	This work
OR.Y7A/Q51A	OR harboring pMU1243; Km^R^; Zeo^R^	This work
OR.W92A	OR harboring pMU1270; Km^R^; Zeo^R^	This work
OR.K144E	OR harboring pMU1276; Km^R^; Zeo^R^	This work
OR.K145E	OR harboring pMU1250; Km^R^; Zeo^R^	This work
OR.Δ121–135	OR harboring pMU1236; Km^R^; Zeo^R^	This work
OR.Δ135–147	OR harboring pMU1251; Km^R^; Zeo^R^	This work
OR.Δ143–156	OR harboring pMU1235; Km^R^; Zeo^R^	This work
OR.Δ152–156	OR harboring pMU1232; Km^R^; Zeo^R^	This work
*E*. *coli*		
DH5α	DNA recombinant methods	Life Technologies
Top10	DNA recombinant methods	Life Technologies
BL21(DE3)	λDE3, T7 RNA polymerase	Life Technologies
XL10-Gold	DNA recombinant methods	Life Technologies
Plasmids		
*Cloning/overexpression*		
pET-15b	Protein overexpression vector; Amp^R^	Novagen
pMU1254	pET-15b harboring *blsA*; Amp^R^	This work
pMU1255	pET-15b harboring *blsA*:Q51A; Amp^R^	This work
pMU1256	pET-15b harboring *blsA*:Δ121–135; Amp^R^	This work
pMU1257	pET-15b harboring *blsA*:Δ135–147; Amp^R^	This work
pMU1258	pET-15b harboring *blsA*:Δ143–156; Amp^R^	This work
pMU1259	pET-15b harboring *blsA*:K145E; Amp^R^	This study
pMU1271	pET-15b harboring *blsA*:Y7A/Q51A; Amp^R^	This work
pMU1264	pET-15b harboring *blsA*:Δ152–156; Amp^R^	This work
pMU1273	pET-15b harboring *blsA*:Y7A; Amp^R^	This work
pMU1282	pET-15b harboring *blsA*:K144E; Amp^R^	This work
pMU1298	pET-15b harboring *blsA*:W92A; Amp^R^	This work
pMU1299	pET-15b harboring *blsA*:F32N; Amp^R^	This work
*Complementation*		
pMU368	*A*. *baumannii-E*. *coli* shuttle vector; Km^R^; Zeo^R^	[20]
pMU1202	pMU368 harboring *blsA* from 17978; Km^R^; Zeo^R^	This work
pMU1213	pMU368 harboring *blsA*:F32N; Km^R^; Zeo^R^	This work
pMU1222	pMU368 harboring *blsA*:A29S; Km^R^; Zeo^R^	This work
pMU1226	pMU368 harboring *blsA*:Q51A; Km^R^; Zeo^R^	This work
pMU1229	pMU368 harboring *blsA*: Y7A; Km^R^; Zeo^R^	This work
pMU1232	pMU368 harboring *blsA*:Δ152–156; Km^R^; Zeo^R^	This work
pMU1235	pMU368 harboring *blsA*:Δ143–156; Km^R^; Zeo^R^	This work
pMU1236	pMU368 harboring *blsA*:Δ121–135; Km^R^; Zeo^R^	This work
pMU1243	pMU368 harboring *blsA*:Y7A/Q51A; Km^R^; Zeo^R^	This work
pMU1250	pMU368 harboring *blsA*:K145E; Km^R^; Zeo^R^	This work
pMU1251	pMU368 harboring *blsA*:Δ135–147; Km^R^; Zeo^R^	This work
pMU1270	pMU368 harboring *blsA*:W92A; Km^R^; Zeo^R^	This work
pMU1276	pMU368 harboring *blsA*:K144E; Km^R^; Zeo^R^	This work

^a^Amp^R^, ampicillin resistance; Km^R^, kanamycin resistance; Zeo^R^, zeocin resistance.

### DNA procedures

Plasmid DNA was isolated from *E*. *coli* cells using a commercial kit (Qiagen). *A*. *baumannii* total DNA was isolated using a phenol-based method described previously [21]. DNA polymerases and restriction enzymes were used according to the manufacturer’s protocols (New England Biolabs). Custom-designed primers (Integrated DNA Technologies) or kit-supplied M13 or T7 primers (Life Technologies) were used to perform all sequencing reactions using BigDye-based chemistry (Applied Biosystems) prior to subcloning reactions and DNA transformations. All primers used in this work are listed in S1 Table.

### *blsA* mutagenesis

**Fig 1:**
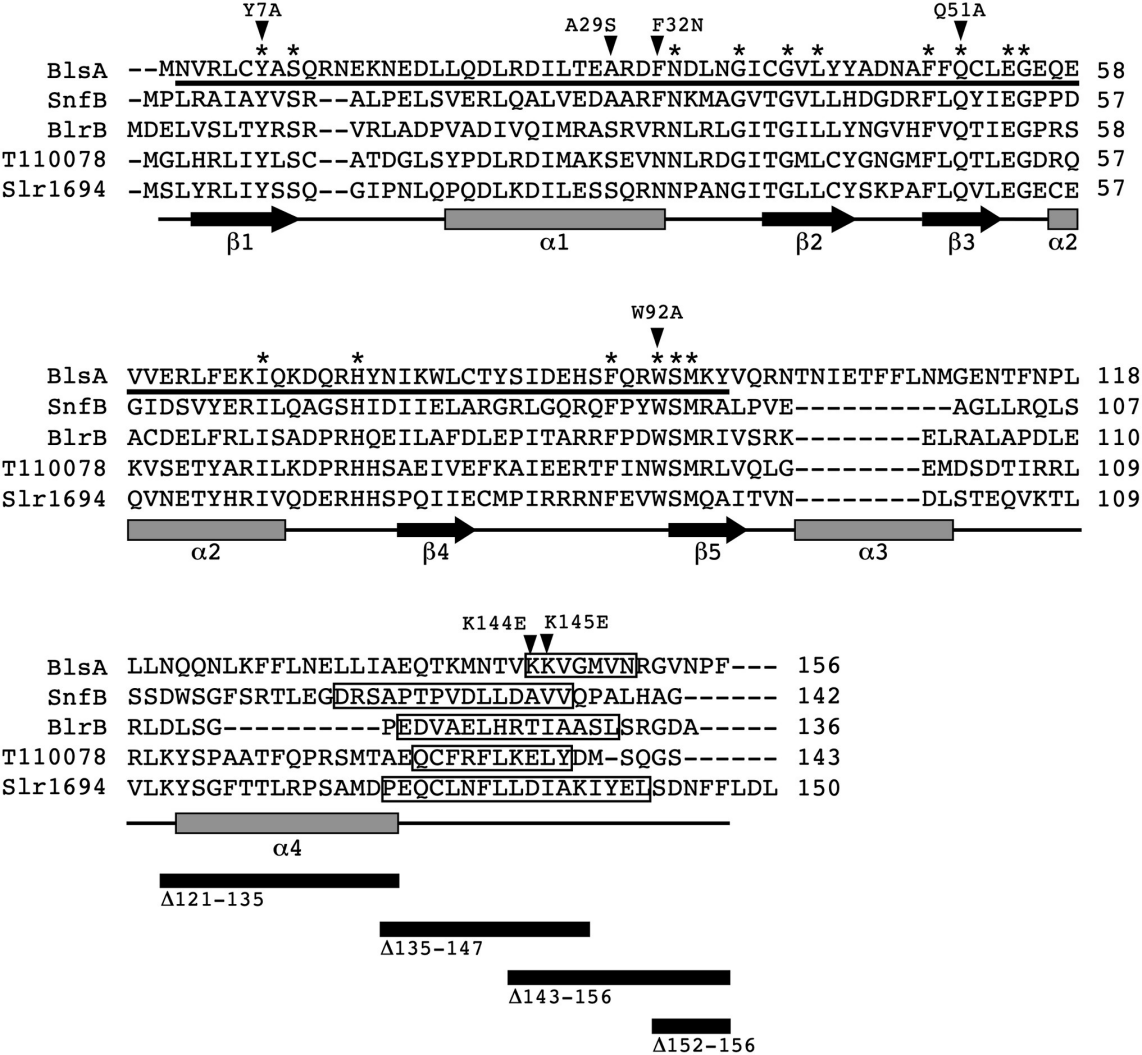
Comparative analysis of short BLUF-containing photoreceptors. The amino acid sequence of the BlsA, SnfB, BlrB, Tll0078 and Slr1694 proteins from *A*. *baumannii* ATCC 17978, *Stenotrophomonas* sp. SKA14, *Rhodobacter sphaeroides*, *Thermosynechoccus elongatus* BP-1 and *Synechocystis* sp. PCC6803, respectively, were compared using MUSCLE. Asterisks identify conserved residues. BlsA predicted secondary structure components are represented by black arrows (β strands) and grey rectangles (α helices). BlsA’s secondary structure was predicted using the SABLE server. The black horizontal bar identifies BlsA’s BLUF domain residues. Black triangles and the corresponding numbers above them indicate site-directed amino acid changes. The boxed BlsA residues identify an additional α component (α_5_) predicted using Robetta. The boxed SnfB, BlrB, Tll0078 and Slr1694 residues identify their cognate α_4_ components. The black horizontal rectangles shown at the bottom of the figure represent locations of site-directed deletions.

The 760-bp chromosomal region encompassing the *blsA* promoter and coding sequences was amplified with Q5 DNA polymerase and primers 4379 and 4380, each flanked with *Bam*HI restriction sites, digested with *Bam*HI and then ligated into the *Bam*HI site of pMU368, a pMAC *A*. *baumannii-E*. *coli* shuttle vector derivative [20], generating pMU1202. Conserved and non-conserved amino acids of the BlsA NT region were targeted by site-directed mutagenesis with the QuikChange Lightning Multi (QCLM) kit (Agilent) using pMU1202 as template and primers 4388, 4392, 4394, 4396, and 4426 to generate the six corresponding derivatives: A29S (pMU1222), F32N (pMU1213), Q51A (pMU1226), Y7A (pMU1229) and W92A (pMU1270), respectively. Primers 4394 and pMU1229 were used to generate a Y7A/Q51A double point mutant (pMU1243). BlsA’s CT point mutations were generated following the same protocol as described for the NT mutagenesis using pMU1202 as template and primers 4425 and 4478 to generate two cognate derivatives: K145E (pMU1250) and K144E (pMU1276). Two CT deletion mutations were generated by inverse PCR using Q5 DNA polymerase and pMU1202 as template with primer pairs 4383/4385 and 4384/4385 to produce pMU1232 (Δ152–156) and pMU1235 (Δ143–156) harboring deletions of the 5- and 14-most CT residues, respectively (Fig 1).

Primers 4386 and 4387 and plasmid pMU1202 were used to generate pMU1236 (Δ121–135) harboring an in-frame deletion of residues located in BlsA’s predicted α_4_ component (Fig 1). Primers 4421/4423 and pMU1202 were used to generate pMU1251 harboring the in-frame deletion of BlsA’s CT residues 135–147 (Δ135–147) shown in Fig 1.

### Detection of surface motility

Cells of the OR *blsA* insertion mutant were electroporated with the pMU368 derivatives listed in Table 1 as described before [22]. Transformants were selected on LB agar containing 100 μg/ml zeocin (Zeo). The hot-Triton plasmid isolation method [23] and agarose gel electrophoresis [19] were used to confirm the presence of complementing plasmids in 17978 derivatives. For surface motility assays, fresh bacterial cultures of each strain were touch-inoculated onto the surface of SA plates incubated at 24°C for 24 h under darkness or light using a blue light LED array (peak emission wavelength, 469 nm) as described previously [9]. Motility assays were performed with three independent fresh bacterial samples in triplicate for each tested sample (*n* = 9). The ImageJ image-processing program (National Institutes of Health) was used to measure the surface motility area of each replicate.

### Overexpression and purification of BlsA and BlsA derivatives

Q5 DNA polymerase and primers 4427 and 4428 were used to PCR-amplify the parental *blsA* coding region from 17978 genomic DNA. The 480-bp amplicon, flanked with *Nde*I and *Bam*HI restriction sites, was ligated into the cognate restriction sites of pET-15b (Novagen), generating pMU1254, which codes for a His-tagged derivative of the parental BlsA protein. Clones harboring *blsA* mutations were PCR-amplified from pMU1213, pMU1226, pMU1229, pMU1243 and pMU1270 using primers 4427 and 4428, as described above. The resulting amplicons were cloned into pET-15b to generate the cognate His-tagged BlsA derivatives harboring NT amino acid mutations. Corresponding plasmids are listed in Table 1 as pMU1299, pMU1255, pMU1273, pMU1271 and pMU1298, respectively. BlsA derivatives with CT deletions were generated by inverse PCR using pMU1254 as a template and primer pairs 4439/4440 and 4439/4441 to generate two pET-15b derivatives: pMU1258 and pMU1264 harboring deletions of residues 143–156 and 152–156, respectively. Inverse PCR using pMU1254 as a template and primer pairs 4386/4387 and 4421/4423 were used to generate two pET-15b derivatives: pMU1256 and pMU1257 harboring in-frame deletions of residues 121–135 and 135–147, respectively. The BlsA CT point-mutation derivatives in pET-15b were constructed by subcloning the *blsA* derivatives from pMU1276 and pMU1250 into pET-15b, as described for the construction of pMU1254, to generate the derivatives pMU1282 and pMU1259, which code for the BlsA K144E and K145E derivatives, respectively. Proper plasmid construction was verified by automated DNA sequencing using T7 primers. A single colony of *E*. *coli* BL21(DE3) transformants harboring one of the pET-15b derivatives that grew on LB agar containing 150 μg/ml ampicillin (LB-Amp) was inoculated into LB-Amp broth, and incubated at 37°C. Overnight cultures were diluted 100 times into 500 mL of LB-Amp and incubated with shaking at 30°C to an OD_600_ of 0.7–0.8. The temperature was then lowered to 18°C, IPTG was added to a final concentration of 0.8 mM, and cells were cultured at 18°C for 15 h in the dark. Cell pellets were resuspended in lysis buffer (20 mM Tris, pH 8.0; 500 mM NaCl; 20 mM imidazole; 1 mM 2-mercaptoethanol [2-ME] and 20 mg/ml DNase and RNase) containing cOmplete EDTA-free protease inhibitor cocktail tablets (Sigma) and lysed with a French Press. Cell debris was removed by ultracentrifugation at 100,000 *g* for 90 min at 4°C. Debris-free lysates were loaded onto a 5-mL HisTrap column (General Electric Healthcare, GE) and washed with 5 vol. of lysis buffer without 2-ME. His-BlsA and derivatives were eluted using an imidazole step-gradient ranging from 20 to 500 mM. His-tagged BlsA and derivatives dissolved in 20 mM Tris, pH 8.0; 500 mM NaCl were further purified and characterized by size-exclusion chromatography (SEC) on an ÄKTA instrument using a Superose 6 Increase 10/300 GL column (GE Healthcare), at a flow rate of 0.5 ml/min. Protein concentration and purity were determined by Bradford assays [24] and SDS-PAGE analysis [25], respectively.

### Spectroscopy analyses

Pure protein samples in 20 mM Tris-HCl (pH 8.0) containing 500 mM NaCl were analyzed with an EPOCH/^2^ microplate reader/spectrophotometer (BioTek) or a Perkin Elmer Lambda 35 UV/Vis spectrophotometer. Protein samples were kept in the dark at 4°C for dark-adapted BlsA samples (dBlsA) before blue-light illumination, with light intensities ranging between 20 and 200 μmol/m^2^/s, for 3 min at 22°C and read using a spectral scan of 1 nm. The spectral absorbance properties of all derivatives were recorded at least twice from two different purified protein samples. For dark state recovery kinetics, purified samples were exposed to blue light for 3 min at 22°C and spectral scans were recorded at 505 nm for 20 min.

### Circular dichroism (CD)

Protein samples dissolved to a final concentration of 3 μM in 10 mM phosphate buffer, pH 8.0 containing 20 mM NaCl were analyzed with an Aviv model 435 CD spectrophotometer at 20°C using a 0.1 cm quartz cuvette. Scans of dBlsA and light-adapted BlsA (lBlsA) were collected at a speed of 500 nm/min between 190 nm and 260 nm. The data are representative of two protein samples run at least twice under the described experimental conditions.

### Chromophore detection

A protocol based on the method described before [26] was used to detect flavin bound to BlsA. Briefly, a 40-μM BlsA sample dissolved in 20 mM Tris, pH 8.0; 500 mM NaCl was incubated at 90°C for 10 min followed by centrifugation at 20,000 *g* for 15 min at 4°C. The supernatant was analyzed by HPLC with an Agilent 1100 LC instrument and a Waters Symmetry C18 reversed-phase column (100Å, 5 μm, 4.6 mm X 150 mm) at room temperature. The column was equilibrated with 50 mM sodium acetate (pH 5.0) containing 5% methanol, and a linear gradient of 5–70% methanol at a flow rate of 0.5 ml/min was used to elute the flavins, which were detected at a wavelength of 450 nm. Standards were made using 100 μM solutions of FAD, flavin mononucleotide (FMN) and riboflavin (Ribo).

### Bacterial growth analysis

Fresh bacterial cultures of the 17978 parental strain and selected derivatives were grown on LB agar plates, inoculated into LB broth supplemented with appropriate antibiotics and cultured overnight at 37°C in a shaking incubator. Cultures were diluted 1:100 into 50 mL of fresh SB without antibiotics and incubated under darkness as described above with constant shaking at 150 rpm in an incubator set at 24°C. OD_600_ reads were taken hourly for 12 h and then at 24 h after inoculation using two independent biological samples.

### Detection of BlsA in *E*. *coli* BL21 cells

Whole-cell lysates of overnight cultures of *E*. *coli* BL21 harboring pET-15b derivatives coding for parental BlsA or mutant derivatives were prepared and size-fractionated by SDS-PAGE using 4%-20% polyacrylamide gradient gels (Bio-Rad) as described before [27]. Proteins were detected by staining with Coomassie Brilliant Blue or western blotting with anti-BlsA antiserum. Protein A labeled with horseradish peroxidase was used to detect the immunocomplexes [28]. Polyclonal anti-BlsA antibodies were generated by immunizing a female New Zealand white rabbit with purified protein as described before [27]. The polyclonal serum was pre-adsorbed with *E*. *coli* and *A*. *baumannii* 17978.OR cell extracts to reduce non-specific reactions as described previously [29]. The immunization protocol was carried out as approved by the Miami University Institutional Animal Care and Use Committee.

### Bioinformatic methods

Amino acid sequences were aligned and compared using EMBOSS Needle and MUSCLE, respectively. The secondary structure of BlsA was determined using the SABLE server. The primary amino acid sequences of short BLUF proteins used for alignments and modeling were downloaded from the Protein Data Bank (PDB). Additional modeling and graphics analysis was performed using the full-chain protein structure prediction server Robetta [30]. The Modeller9v8 was also used to generate a 3D model of BlsA [31, 32]. The stereochemical quality of the 3D model was assessed using a Ramachandran plot.

The BlsA model produced by the Robetta server was superimposed with 0.853 Å rmsd for all backbone atoms with chain A of the AppA structure deposited with PDB ID 2iyg [33]. We extracted the coordinates for the flavin from 2iyg and docked the flavin into the BlsA model followed by minor side chain adjustments using Coot [34]. The docked flavin was easily accommodated within the BlsA model with the primary adjustment being reorientation of the E28 side chain to a torsion similar to the preferred **mm**-40° rotamer with 8.66% probability [35]. Building of the BlsA model with bound flavin, the K144E and K145E mutations were generated *in silico* using Coot. Both K144E and K145E were easily accommodated with the preferred **mt**-10° rotamer with only minor adjustments needed for K145E. Electrostatic surface maps were prepared using the BlsA wild type, K144E, and K145E models as input into PDB2PQR [36, 37] and APBS [38, 39] with visualization in PyMOL (The PyMOL Molecular Graphics System, Version 2.3.0 Schrödinger, LLC).

### Statistics

The statistical significance of collected experimental data was analyzed by the student’s *t-*test or analysis of variance (ANOVA) using the GraphPad Prism version 7.0d for Max OS X (GraphPad Software). *P* values ≤ 0.05 were considered statistically significant for all aforementioned assays. Error bars represent the standard error for each data set shown in the figures.

## Results and discussion

### Overall predicted BlsA structure

**Fig 2:**
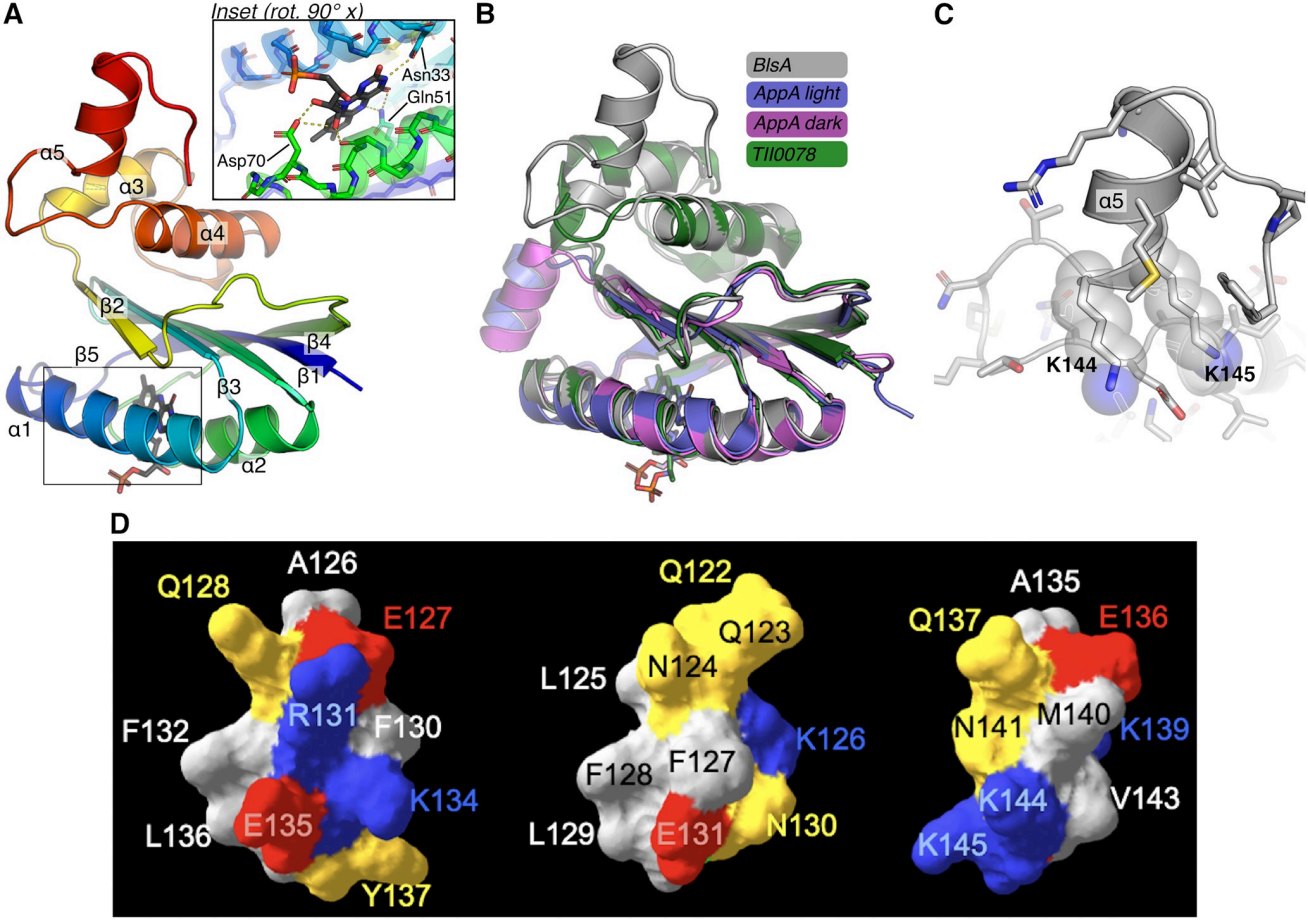
Structural model of BlsA and comparison to known blue light sensing domains. (A) Model of BlsA built using Robetta. BlsA is drawn as a cartoon colored by spectrum from the N-terminus (blue) to C-terminus (red) with secondary structure elements labeled and FAD drawn as sticks. The zoomed inset (rotated ~90° about the *x*-axis) shows BlsA residues (sticks) N33, Q51 and D70 making electrostatic contacts (yellow dashes) with FAD. (B) The BlsA model is superimposed with the structure of the AppA BLUF domain in the light- (blue, PDB ID 1yrx) and dark- (purple, PDB ID 2iyg) adapted states and Tll0078 (green, PDB ID 1x0p). (C) A zoomed view of BlsA helix α_5_ from the Robetta model highlights the positions of K144 and K145. (D) Surface model of the Tll0078 α_4_ (left) compared with surface models of two predicted BlsA CT α helices (middle and right) drawn using Modeller9v8. Yellow (polar), red (negative), blue (positive), grey (nonpolar). Visible residues are labeled according to their position in the amino acid sequence of the cognate proteins.

The class of short BLUF-containing photoreceptors, which is the main focus of this work, includes small proteins such as Tll0078 from *T*. *elongatus* BP-1 [40], Slr1694 from *Synechocystis* sp. PCC6803 [16], SnfB from *Stenotrophomonas* sp. SKA14 [41], BlrB from *R*. *sphaeroides* [42] and the BlsA protein we identified in *A*. *baumannii* ATCC 17978 [9]. The comparative analysis of these proteins showed that the amino acid sequence of BlsA and Tll0078 are 26% identical and 39.9% similar, whereas BlsA and Slr1694 share 30.7% and 47.6% identity and similarity levels, respectively. The identity and similarity between BlsA and BlrB are 23% and 36%, respectively, while BlsA and SnfB share 21.7% and 41.6% identity and similarity indices, respectively. The amino acid sequence alignments also revealed that all 16 residues identical in these five proteins are located within their BLUF domains, that in BlsA encompasses residues 2–96 (Fig 1). In contrast, there were no conserved residues outside the BLUF domains among these proteins. These features reflect the general observation that light sensing by short photosensors is mediated by the interaction of the FAD chromophore with critically conserved as well as semi-conserved BLUF domain residues, while signal transduction is mediated by CT terminal residues and amino acid sequences that are not conserved and do not show significant similarity to known proteins, respectively [17]. The secondary structure of BlsA is also predicted to be comparable to those reported for Tll0078, Slr1694, BlrB and SnfB (S1 Fig). In the case of Tll0078 [18], Slr1694 [43] and BlrB [42] these predictions were confirmed by crystallography studies, which showed that the β_1_α_1_β_2_β_3_α_2_β_4_β_5_ and α_3_α_4_ structures located at their BLUF domains and CT terminal regions, respectively, are a common structural feature of short FAD-dependent photosensing proteins. Based on all these similarities, we constructed the predicted tertiary structure of the entire 156-amino acid BlsA sequence, which is displayed in Fig 2A and 2B. Although this model shows the typical β_1_α_1_β_2_β_3_α_2_β_4_β_5_α_3_α_4_ structure described in BLUF proteins, the comparative analysis with Tll0078 and the light- and dark-adapted AppA BLUF domain shows that their predicted structures superimpose with the exception of the additional α helix (α_5_) formed by the BlsA KKVGMVN residues, which are located between positions 144–150 (Figs 1 and 2C).

The prediction of the α_5_ component is supported by additional modeling, which shows that the surface topology of the BlsA region defined by residues A135-K145 is similar to that displayed by the Tll0078 residues A126-Y137 that map within the predicted α_4_ component of this protein (Figs 1 and 2D). The structural assessment of the 3D model quality revealed that > 94% of these residues are in the most favored regions as evidenced by the Ramachandran plot. The biological effects of the K144E and K145E point mutations described later in this report lend further support to the proposed model, which includes the NT FAD-binding photoreceptor domain and a CT domain with an additional α component that could be involved in signal transduction upon illumination.

Taken together, these observations indicate that although the overall BlsA structure is highly similar to other short BLUF-containing photoreceptors, it may have unique structural properties that would explain some of the distinct observations described in this report.

### Analysis of the BlsA N terminus

#### Functional studies

**Fig 3:**
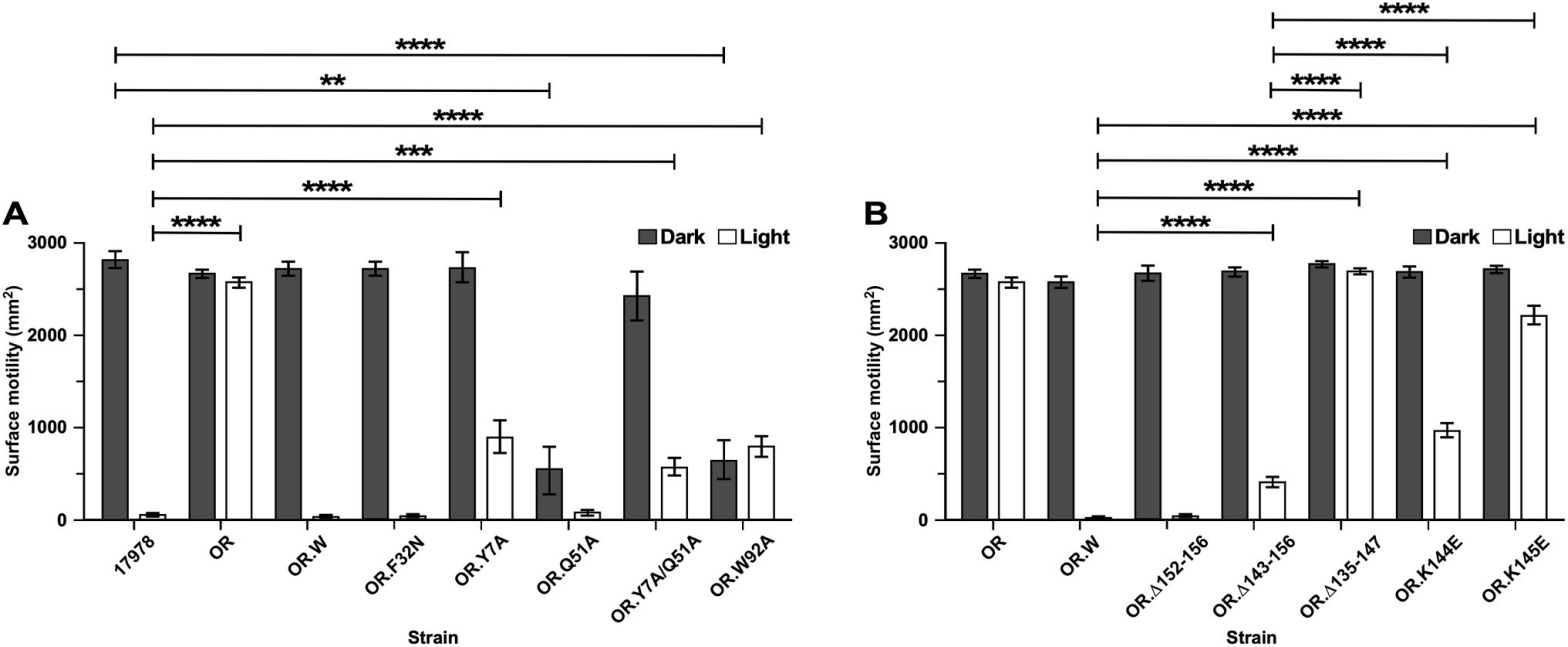
Light-regulated surface motility of 17978 cells producing native BlsA or derivatives generated by site-directed mutagenesis. (A) Motility of OR BlsA-deficient cells transformed with a pMU368 derivative coding for the F32N (OR.F32N), Y7A (OR.Y7A), Q51A (OR.Q51A), Y7A/Q51A (OR.Y7A/Q51A) or W92A (OR.W92A) NT mutant proteins. (B) Motility of OR BlsA deficient cells transformed with a pMU368 derivative coding for the Δ152–156 (OR.Δ152–156), Δ143–156 (OR.Δ143–156), Δ135–147 (OR.Δ135–147), K144E (OR.K144E) or K145E (OR.K145E) CT mutant proteins. The motility responses of the 17978 parental strain (17978), the BlsA OR mutant (OR) and the OR mutant transformed with a pMU368 derivative coding for the wild type protein (OR.W) were used as controls. Surface motility was tested using SA plates incubated at 24°C under darkness or illumination. Horizontal bars identify statistically different values (*P* ≤ 0.01, **; *P* ≤ 0.001, ***; *P* ≤ 0.0001, ****) and error bars represent the standard error of each data set.

Based on sequence identity among BLUF domains located in different photosensor proteins, Brust *et al*. [13] proposed a model highlighting, as a distinct BlsA feature, the presence of an F rather than the N residue found at position 32 in other BLUF proteins, such as Slr1694 (Fig 1). This feature implies that although the BlsA F32 residue does not H-bond to the FAD C2 = O carbonyl group as the Slr1694 N residue does, its presence at a position close to the chromophore moiety suggests that the rearrangement of the H-bonding network in the FAD-binding pocket in response to light may be affected. TRIR spectroscopy studies indicated that changing F32 to H, which is the AppA analogous residue, does not significantly alter the spectra of BlsA exposed to light (lBlsA) or kept under darkness (dBlsA) [13]. In contrast, changing F32 to N, the Tll0078 and Slr1694 analogous residues (Fig 1), results in spectral differences between lBlsA and dBlsA. These differences indicate that the presence of a N residue at position 32 results in stronger H-bonding interactions between BlsA and FAD [13]. These spectroscopy observations prompted us to examine the biological effect, measured as the light-mediated BlsA-dependent surface motility response we described before [9], of the F32N mutation. Fig 3 shows that the motility of the 17978.OR BlsA mutant under illumination at 24°C was significantly increased (*P* ≤ 0.0001) when compared with the response of the 17978 parental strain under the same experimental condition.

Analysis of OR.W cells showed that the electroporation of pMU1202, a derivative of the pMU368 shuttle vector harboring a copy of the parental *blsA* allele expressed from its natural promoter (Table 1), reduced motility under illumination to wild type levels. Such a response was not observed when OR.V cells, which harbored empty pMU368, were tested under the same experimental conditions. Furthermore, restriction analysis of plasmids isolated from the complemented strains demonstrated that pMU368-based complementing vectors were stably maintained as independent replicons without noticeable rearrangements throughout the motility assays, which were conducted using SA without the addition of a selecting antibiotic.

The analysis of OR.F32N cells, expressing BlsA F32N (Table 1), showed that this amino acid change did not alter the motility responses under darkness or illumination when compared with the 17978 and OR.W strains (Fig 3). This observation indicates that although a change at position 32 affects FAD-BlsA interactions according to spectroscopy studies [13], this change does not have a significant impact on BlsA’s biological role. This observation agrees with the finding that F is not a conserved residue at analogous locations among short as well as long BLUF receptors described in different microorganisms [17].

The model proposed by Brust *et al*. [13] predicts that Y7 is H-bonded to Q51 to form the critical FAD-protein association network required for light sensing (Fig 2A inset), which was also described for other BLUF proteins including Slr1694 [16] and Tll0078 [18]. As described for the analogous Tll0078 Y8 and Q50 residues, it is possible to predict that upon light stimulation, BlsA structural modifications allow the Q51 side-chain oxygen to form a H-bond with the hydroxyl oxygen of Y7, while the side-chain amino group of Q51 forms a tight H-bond with the C4 = O of FAD. Thus, the critical roles that Y7 and Q51 could play in BlsA’s biological functions were also tested using motility assays. In contrast to F32N, the 17978.OR complemented derivatives expressing the Y7A, Q51A and Y7A/Q51A mutations (Table 1), showed significantly altered light-regulated motility responses. The OR.Y7A and OR.Y7A/Q51A complemented mutants displayed significantly increased motility under illumination when compared with the response of 17978 (*P* ≤ 0.0001 and *P* ≤ 0.001, respectively) as well as OR.W cells harboring a plasmid copy of the parental allele (Fig 3). The Y7A and Y7A/Q51A residue changes did not significantly alter the motility of these mutants under darkness. Interestingly, although the motility response of the OR.Q51A derivative under illumination was comparable to that displayed by 17978 and OR.W, the Q51A mutation resulted in a significant reduction (*P* ≤ 0.0001) of surface motility under darkness (Fig 3). Notably, the growth curve of the OR.Q51A derivative was not significantly different from that displayed by the parental strain when cultured in SB at 24°C under darkness (S2 Fig). This observation indicates that the reduced surface motility of the Q51A mutant cannot be explained by a simple negative effect on bacterial growth in the absence of light.

The W92 residue, which is located just before the predicted β_5_ strand (Fig 1), could be crucial for BlsA functions based on the conservation of this residue at analogous positions in the aligned amino acid sequences shown in Fig 1. In the case of Slr1694, the analogous W91 residue could move from a hydrophobic to a hydrophilic environment upon illumination, a process that potentially conducts the signal transduction process associated with the response to illumination [43]. These observations prompted us to test the effect of the W92A mutation on light-regulated motility. The significantly enhanced motility phenotype (*P* ≤ 0.0001) of the OR.W92A derivative when compared with 17978 and OR.W upon illumination (Fig 3) supports the role of this amino acid residue in BlsA biological activity, which could be due to the interaction with downstream effectors as proposed by Brust *et al*. [13]. However, as it was observed with Q51A, the W92A mutation significantly reduced (*P* ≤ 0.0001) the dark motility response when compared with 17978 and OR.W (Fig 3), without affecting the overall growth of OR.W92A when compared to both the 17978 parental strain and the OR.Q51A mutant cultured at 24°C under darkness in SB (S2 Fig).

It is apparent that, when compared to all tested strains, the motility of the OR.Q51A and OR.W92A mutants under darkness is significantly reduced to the point that it resembles the response of the 17978 parental strain under illumination. The reduced motility response of OR.Q51A under darkness could be explained by the observation that Q50A and Q63E mutations in the analogous sites of Slr1694 and AppA, respectively, resulted in derivatives locked in pseudo-light-excited states that produced spectral responses mimicking illumination when tested under darkness [44, 45]. Unfortunately, we could not test this hypothesis since the Q51A point mutation, along with other tested N-terminal mutations, resulted in the overexpression of insoluble proteins as described later in this report. Interestingly, the Y7A/Q51A double mutation did not trigger the same motility response as the single Q51A mutation (Fig 3A). Notably, based on our motility data, the W92A BlsA derivative also seems to represent a lit-state derivative (Fig 3A). This observation is in contrast to that produced by the analogous W91A Slr1694 derivative, which displayed wild type photochemistry and signal transduction responses [45]. This observation indicates that although W92 does not directly interact with FAD, it plays a role in the overall BlsA-flavin interactions involved in light sensing.

The BlsA A29 residue, which is located within α_1_ (Fig 1) is different from the analogous S residue of BlrB, Tll0078 and Slr1694. This residue has previously been reported to play a role in the excited state stabilization of the protein. The ground state recovery kinetics of a BlsA A29S mutant compared to the wild type protein increases approximately 2-fold in its lifetime [46]. This phenomenon was only observed for dBlsA. Similar to data obtained for the AppA protein, whose analogous residue is S41, no observable spectroscopy effects were measured for the light-adapted state. To test the biological role of this residue, we generated a BlsA A29S derivative. Motility assays showed that the response of OR.A29S cells (Table 1) was not significantly different from that displayed by the 17978 parental strain in either light or dark conditions. These responses suggest that BlsA’s A29 residue is not essential for its activity, a possibility that is supported by the presence of an analogous A residue in the SnfB photoreceptor (Fig 1).

Taken together, all the observations presented above highlight the fact that BlsA shares some common structural and functional properties with different short BLUF photoreceptors that are produced by unrelated bacteria, yet also exhibits some unique properties of its own.

#### Protein studies

**Fig 4:**
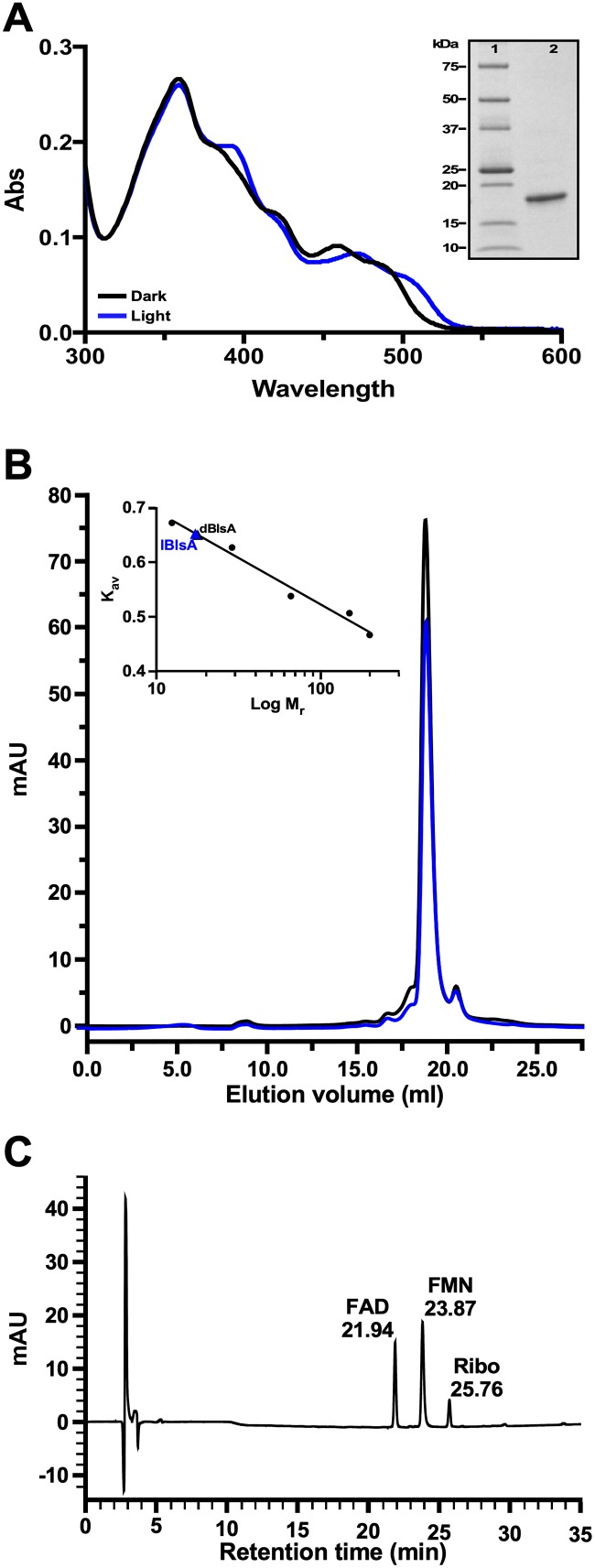
Analyses of His-tagged BlsA. (A) Absorption spectra of lBlsA and dBlsA. The UV-Vis light spectra were recorded using His-tagged BlsA purified by Ni-affinity chromatography and SEC. The purity of the protein sample used for spectral analyses was confirmed by SDS-PAGE using 4%-20% polyacrylamide gradient gels (inset). (B) Elution profile of lBlsA and dBlsA upon chromatography in a Superose 6 Increase 10/300 GL column. The inset shows the calibration curve constructed using the elution volume of the gel filtration standards cytochrome C (12.4 kDa), carbonic anhydrase (29 kDa), bovine albumin (66 kDa), alcohol dehydrogenase (150 kDa) and β-amylase (200 kDa). The black and blue triangles indicate the elution position of dBlsA and lBlsA, respectively. (C) HPLC analysis of heat-denatured BlsA supernatant. The retention times for FAD, FMN and Ribo are indicated in min.

To further characterize the effect of the NT mutations described above, the *blsA* parental allele and the derivatives generated by site-directed mutagenesis were cloned in pET-15b and transformed into *E*. *coli* BL21. IPTG induction of *E*. *coli* BL21 cells expressing His-tagged BlsA resulted in the overproduction of a protein band that reacted with anti-BlsA antibodies (S3 Fig, lane 3 in panels A and B). Although a significant amount of His-BlsA was present in the pellet after ultracentrifugation, the supernatant contained enough recombinant protein that could be isolated for further analysis. The His-tagged BlsA derivative, which was purified to homogeneity by Ni-affinity chromatography and SEC, displayed the characteristic 10-nm red shift upon blue light irradiation (Fig 4A and inset) we reported before [9].

Comparable spectra were produced by protein samples exposed for 3 min at 20, 100 or 200 μmol/m^2^/s blue light intensities (S4 Fig). The spectral analysis also showed that purified lBlsA relaxed back to the dark-adapted state with a t_1/2_ of 10.2 min and a τ rate of 14.7 min (Table 2), values that are comparable to those reported previously [13].

**Table 2:** Kinetic values for light-to-dark recovery of His-BlsA and derivatives generated by site-directed point and deletion mutations.

Protein	Half-time—t_1/2_ (min)	Time constant—τ (min)
His-BlsA	10.2	14.7
His-BlsA.K144E	5.2	7.4
His-BlsA.K145E	7.6	11.0
His-BlsA.Δ152–156	8.6	12.4
His-BlsA.Δ143–156	5.2	7.4

The SEC experiments not only produced pure photoactive BlsA preparations, but also showed that gel filtration chromatography of dBlsA resulted in the identification of a single 17.7-kDa protein fraction, while that of lBlsA produced only one fraction displaying a molecular mass of 17.2 kDa. Both protein masses match the predicted mass of the monomeric protein (Fig 4B and inset). Analysis of different protein preparations produced the same outcome without the detection of dimers or larger BlsA multimers under the experimental conditions used to isolate and analyze overexpressed proteins.

In contrast to the analysis of BlsA, the same overexpression and purification approach failed to produce meaningful results for the Y7A, Q51A, Y7A/Q51A and W92A His-tagged BlsA recombinant derivatives. SDS-PAGE analysis of whole cell lysates of *E*. *coli* BL21 transformants overexpressing the Y7A, Q51A, Y7A/Q51A or W92A BlsA derivatives (Table 1) showed the presence of a protein reacting with anti-BlsA antibodies upon IPTG induction (S3 Fig, lanes 4–7). However, most of the recombinant proteins were present in the pellets collected after ultracentrifugation of cell lysates with little to no His-tagged BlsA derivatives recovered after Ni-affinity column chromatography. Although these results are in line with those observed during the analysis of the *R*. *sphaeroides* AppA flavoprotein, whose proper folding and consequent solubility depend on flavin binding [47], they contrast with the outcome of the overexpression of AppA126 (residues 1–126 harboring the AppA BLUF domain) and the AppA126 W104A mutant, both of which were obtained as soluble recombinant products of pTY-derivatives overexpressed in *E*. *coli* BL21 [48]. Our results with the Q51A mutant also contrast with those of the analogous Tll0078 Q50A derivative, which was overexpressed as a soluble N-His-tagged recombinant product even when *E*. *coli* BL21 cells were incubated at 37°C after IPTG induction [18]. Furthermore, the mutagenesis of the BlrB Y9 and Q52 residues did not impair the overexpression and purification of derivatives that were characterized by their capacity to photocycle and bind FAD [49]. Our ability to obtain a soluble His-tagged BlsA derivative using an overexpressing vector different from the pET-TEV plasmid we used in our initial report [9] and reproduce the outcome reported during the spectroscopic analysis of BlsA [13] strongly indicate that our failure to obtain soluble Y7A, Q51A, Y7A/Q51A and W92A His-tagged derivatives is not due to technical and/or procedural problems.

Taken together, the functional and protein studies described above indicate that the Y7, Q51 and W92 NT residues are critical not only for light sensing, but also BlsA’s stability and/or structural integrity, which seems to depend on its capacity to bind flavins, when overexpressed in a heterologous host. Notably, this is a potentially distinct property of BlsA when compared to related short BLUF recombinant proteins.

### Analysis of BlsA C terminus

Although BLUF domains of bacterial photoreceptors are highly conserved, their C-terminal amino acid sequences are highly divergent. In the case of BlsA, the primary sequence of this region has no significant similarity to known or characterized proteins that could predict its role in BlsA’s overall function. However, as described above, BlsA’s CT region may comprise not only the α_3_α_4_ components found in related short BLUF photosensors, including Tll0078, Slr1694, BlrB and SnfB, but also an additional α_5_ element (Figs 1 and 2A–2C), which encompasses residues 144–150. The absence of this additional predicted α-helical element in related bacterial chromophores may reflect potential structural and functional differences between BlsA and aforementioned BLUF-containing chromophores. It has been suggested that BlsA’s CT amino acids are involved in particular signal transduction and regulatory processes [9, 13, 50, 51], but their function has not been tested experimentally. Based on these observations, we decided to determine whether BlsA’s CT residues play a role in its biological functions.

#### Functional studies

We started by testing the effect of deleting the last five CT amino acid residues on light-regulated motility. Fig 3B shows that the OR.Δ152–156 complemented derivative (Table 1) displays a light-dependent differential surface motility response comparable to that of the 17978 parental strain and the OR.W derivative complemented with the *blsA* parental allele. In contrast, the OR.Δ143–156 BlsA derivative (Table 1) displayed significantly increased (*P* ≤ 0.0001) motility under illumination when compared to the response of the OR.W derivative, although to a much lower level than the OR mutant response (Fig 3B). The most drastic change in surface motility responses upon illumination (*P* ≤ 0.0001), which reached the levels displayed by the OR strain, was detected with the complemented derivative OR.Δ135–147 that expresses the BlsA:Δ135–147 in-frame deletion mutation (Fig 3B). Taken together, these results indicate that the region located between residues A135 and N150, which includes our predicted α_5_ component (Figs 1 and 2A), is critical for BlsA functions. This possibility is further supported by the observation that OR.K144E and particularly OR.K145E cells displayed significantly increased surface motility responses (*P* ≤ 0.0001) under illumination when compared to OR.W (Fig 3B). The motility phenotype of the OR.Δ121–135 derivative, which was comparable to that displayed by the OR BlsA mutant under darkness or illumination, indicates that in-frame deletion of the α_4_ component causes significant structural changes that impair BlsA’s biological functions.

#### Protein studies

**Fig 5:**
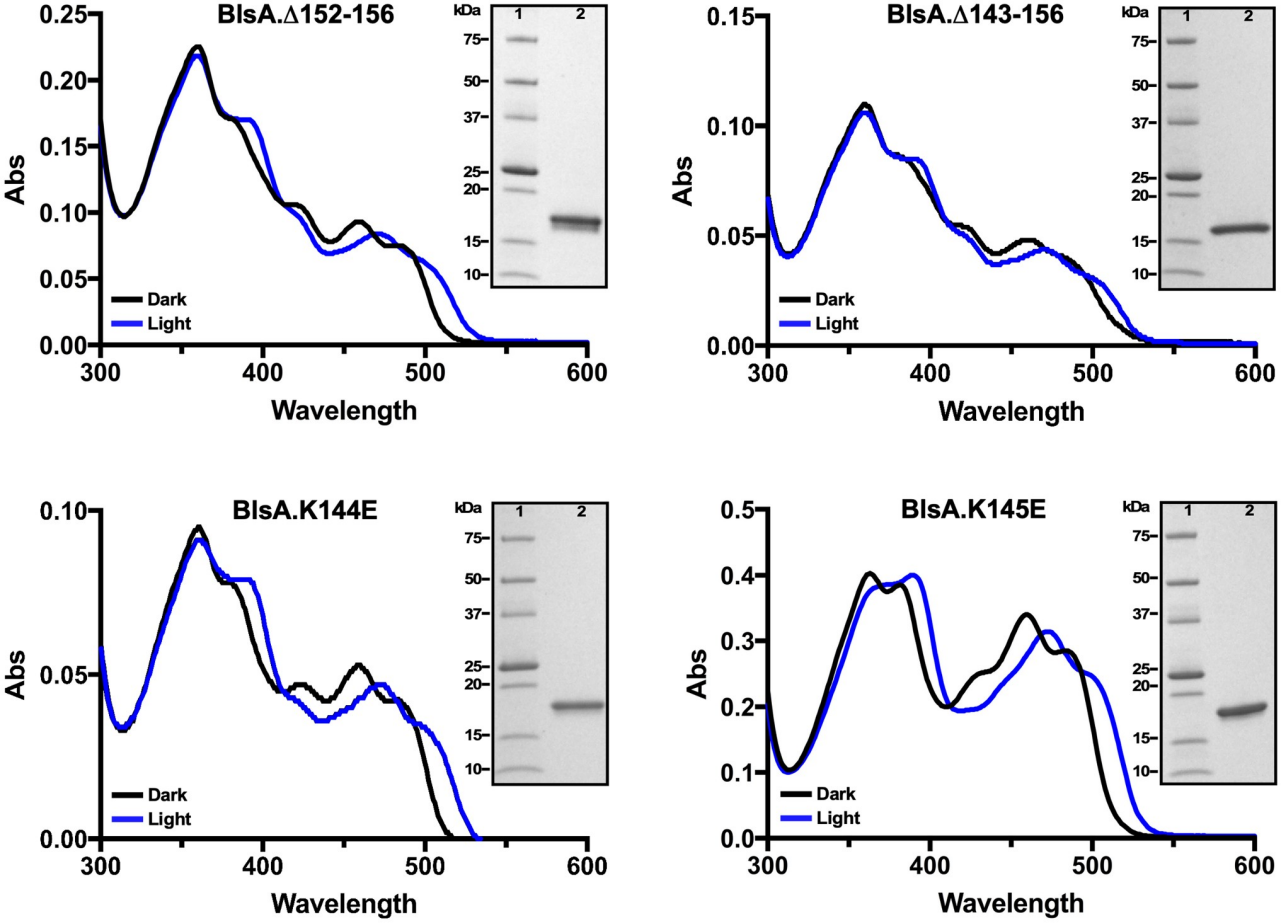
Spectral analysis of purified His-tagged BlsA derivatives generated by site-directed deletion and point mutagenesis. The UV-Vis light spectra were recorded using His-tagged BlsA derivatives purified by Ni-affinity chromatography and SEC. The purity of the protein samples used for spectral analyses was confirmed by SDS-PAGE using 4%-20% polyacrylamide gradient gels (insets).

*E*. *coli* BL21 cells overexpressing the Δ152–156, Δ143–156, K144E or K145E derivatives produced readily visible yellow-stained homogenous protein fractions upon Ni-affinity and size-exclusion chromatography, the latter of which yielded data indicative of only protein monomers as it was observed with the His-tagged BlsA recombinant product. When analyzed by SDS-PAGE, these samples displayed the predicted molecular sizes (Fig 5 insets).

The top panels of Fig 5 also show that the deletion of neither the last 5 (BlsA.Δ152–156) nor 14 (BlsA.Δ143–156) CT amino acid residues significantly affected the photoresponses of these BlsA recombinant derivatives. Both of these derivatives displayed a red shift similar to that of the His-BlsA derivative upon illumination (compare panel A in Fig 4 with top panels in Fig 5). However, it is worthy to note that complementation of OR with *blsA*:Δ152–156 but not with *blsA*:Δ143–156 fully restored the parental light-regulated motility response (Fig 3B). Although motility responses of the OR.K144E and OR.K145E complemented strains, particularly the latter mutant, were significantly impaired when compared with the response of the OR.W strain (Fig 3B), spectral analyses showed that the K144E and K145E point mutations did not affect the photoresponse of these BlsA derivatives upon blue light illumination as reflected by a red shift similar to that displayed by the His-BlsA derivative (compare panel A in Fig 4 with bottom panels in Fig 5).

In contrast to the spectral observations, analysis of recovery kinetics showed that the four BlsA CT mutations described above impacted this BlsA property when compared to the His-BlsA derivative. Table 2 shows that the K144E and Δ143–156 point and deletion mutations accelerated light-to-dark reversion kinetics to τ values of 7.4 min, rates that represent an almost 2-fold change when compared with His-BlsA. For the K145E (τ = 11.0 min) and the Δ152–156 (τ = 12.4 min) mutations, the reversion kinetics rates were accelerated by 1.3- and 1.2-fold, respectively, when compared to the BlsA His-tagged parental derivative.

These observations indicate that residues located between positions 143 and 151, including K144 and K145, play a role in BlsA regulatory but not FAD-dependent photoresponse. This possibility is supported by the spatial location of these residues afar from the amino acid network directly interacting with the FAD chromophore (Fig 2A). However, our data indicate that CT residues could play a role in the photodynamic properties of BlsA, although to a different degree as in the case of residues K144 and K145, by molecular mechanisms that remain to characterized.

Overexpression of BlsA.Δ121–135 or BlsA.Δ135–147 (Fig 1) in *E*. *coli* BL21 also produced His-tagged BlsA derivatives that were found predominantly in the cell pellets after ultracentrifugation. These results suggest that the deletion of the α_4_ component or the adjacent 12-residue region, which includes the KKVG N-terminal residues of the putative α_5_ component (Figs 1 and 2A), alters BlsA stability. This outcome is most likely due to significant changes in folding of BlsA that result in insoluble or aggregated proteins when overexpressed in *E*. *coli* cells upon IPTG induction. The insolubility of these BlsA derivatives as well as those affecting the NT region of this protein must be taken into consideration in the analysis of the motility responses of bacteria expressing them.

Taken together, the motility and protein results indicate that although the last five CT residues are functionally dispensable, at least as measured by the experiments we have outlined, residues located between positions 135 and 151 are critical in downstream signal transduction without playing a major role in FAD-mediated light sensing processes.

#### CD spectroscopy

The potential effects of the point and deletion mutations described above on the predicted secondary structure and folding properties of BlsA and its derivatives were assessed by CD spectroscopy. This analysis showed that the spectra of dBlsA and lBlsA His-tagged derivatives were comparable and compatible with a protein harboring the α-helix and β-sheet components predicted to be present in BlsA’s tertiary structure (S5 Fig). This analysis further showed that the spectra of the dark- and light-adapted BlsA.K144E, BlsA.K145E, BlsA.Δ152–156, and BlsA.Δ143–156 point mutation and deletion derivatives, respectively, are not significantly different from each other (S5 Fig). This outcome is further supported by the observation that none of these mutations resulted in predicted secondary structures significantly different from that shown in Fig 1 for the parental BlsA protein. Taken together, these results suggest that the biological effects of these four BlsA mutations cannot be attributed to drastic changes in the structure of encoded proteins.

#### Chromophore analysis

The capacity of BlsA to bind flavins was determined by HPLC analysis, which showed that the supernatant of heat-denatured His-tagged BlsA contains components with the same retention times displayed by the FAD, FMN and riboflavin standards (Fig 4C and S6 Fig). It is apparent that FAD and FMN are the major flavin components bound to BlsA with riboflavin representing a minor component. The presence of these flavins in purified BlsA could be due to the replacement of FAD with FMN and the hydrolysis of the later flavin during overexpression in *E*. *coli* BL21, a phenomenon that was reported for the SnfB photoreceptor [41] and the *R*. *sphaeroides* AppA BLUF domain [52].

The same HPLC analysis of the K144E, K145E, Δ152–156 and Δ143–156 BlsA derivatives showed that neither the residue substitutions at positions 144 and 145 nor the deletion of the last 5 and 14 amino acid residues impaired the binding of the flavin components listed above by the BlsA NT region (S7 Fig). This observation is congruent with the fact that the responses of these proteins to illumination are comparable among themselves (Fig 5) and with the His-tagged BlsA recombinant protein (Fig 4).

## Conclusions

The light-sensing domain of long BLUF sensor proteins is associated with effector domains that demonstrate recognizable functions including those involved in the biosynthesis and degradation of cyclic di-GMP (diguanylate cyclases and phosphodiesterases, respectively), biosynthesis of cyclic AMP by adenylyl cyclases, transcriptional control of gene expression conferred by helix-turn-helix DNA binding motifs or a sensor containing a heme instead of cobalamin (SCHIC) domain. In contrast, the BLUF domain of short photosensors is associated with relatively short amino acid sequences that cannot be used to predict biological/biochemical functions [53]. While the work published on the analysis of short bacterial photoreceptors has provided critical information on structural and spectroscopic properties of these proteins, not much has been published on structure-function relationships that play a role in the biological functions of these proteins. Our work has revealed unexplored BlsA structure-function relationships that could play a role in the sensing and regulatory roles of this *A*. *baumannii* protein. The hydrogen-bonding network around the FAD chromophore, including the conserved Y7 and Q51 and the semiconserved W92 residues, is critical for *A*. *baumannii* light sensing; differential expression of light-controlled functions, such as those involved in the interaction with semisolid surfaces as we previously reported [9, 54]; and protein stability when overproduced in an unrelated bacterial host. Replacement of Y7 and Q51 by A residues should abolish the interactions needed not only for electron- and proton-transferring activities, but also for BlsA-flavin interactions required for proper protein folding and stability. However, it is yet-to-be-determined whether the motility results reflect the inability of the NT mutants to bind FAD and sense light and/or their stability/solubility properties. The motility data collected with the Q51A and W92A mutants, both of which display a lit-state (Fig 3A), suggest that the motility responses of these two mutants are most likely not due to protein insolubility/stability. The motility response of these two mutants should be comparable to the response of the OR BlsA mutant incubated under darkness if the Q51A and W92A BlsA derivatives produce insoluble proteins.

The effect of the BlsA W92A mutation could be explained by the role the analogous W104 residue plays in the photochemistry of the AppA BLUF domain; the interaction of this residue with Q63, which is analogous to the BlsA Q51 residue, is critical for AppA light-sensing functions [55]. Notably, Slr1694 activity depends on the interaction of Q50, which is analogous to the BlsA Q51 residue, with an M residue located at position 93 of the Slr1694 protein [56], a residue that is conserved in analogous locations of the BLUF proteins aligned in Fig 1. Taken together, these observations indicate that BlsA is functionally more similar to the BLUF domain of the long AppA photoceptor than to the Slr1694 short photoreceptor, although their photochemistry involving the flavin chromophore is similar.

Our analysis of the BlsA CT region also provided critical information. The last five amino acid residues may represent a non-functional region since this set of residues plays a role in neither BlsA’s structural integrity nor its photochemical and gene regulatory functions, at least as measured by regulation of surface motility in response to light. In contrast, the region limited by residues A135 and R151, which is located immediately after the α_4_ component and includes the first two N-terminal residues of the predicted α_5_ element (Fig 1), proved to be critical for the expression of light-regulated motility functions as well as the stability of overexpressed protein in a heterologous host. On the other hand, the region encompassing the last 14 residues appear to be critical for the regulation of light-mediated responses without having a significant role in protein stability and flavin biding. These observations do not resemble those collected using BlrB CT truncated derivatives [49]. Although the BlrB 122 and BlrB 130 deletions, which ended at the first residue and in the middle of the α_4_ element predicted by NMR, respectively, resulted in proteins that were expressed, the flavin binding capacity of these derivative proteins was significantly impaired. The BlrB 135 deletion, which removed the last three protein residues and ended two residues after α_4_, resulted in a less stable derivative, when compared with the full protein, that was nonetheless capable of binding the flavin chromophore. Taken together, these observations suggest that although the amino acid sequence and structure of BlsA and BlrB are comparable, each have distinct functional properties.

**Fig 6:**
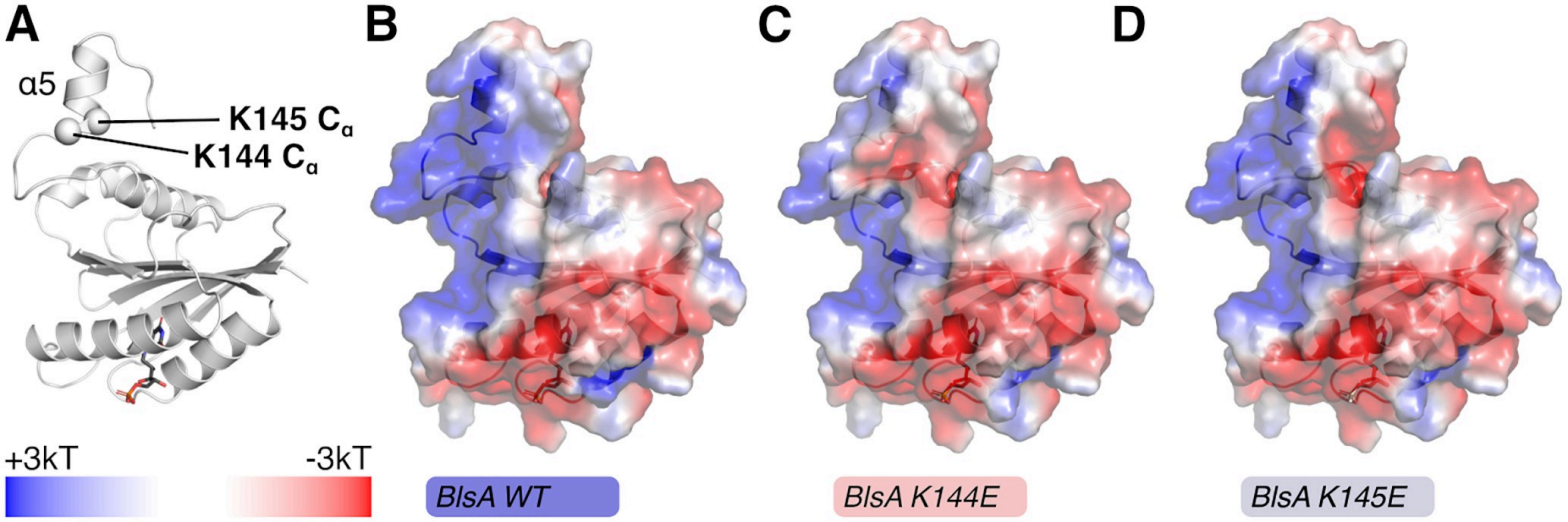
Effects of K144E and K145E mutations on the electrostatic surface of BlsA. (A) Cartoon model of BlsA, built using Robetta, with the positions of the ⍺-carbons of K144 and K145 shown as spheres. The color bar indicates correspondence for the electrostatic surfaces shown in panels B-D between color and charge at ±3kT. Panels B, C and D show the surfaces for wild type BlsA and the K144E, K145E derivatives, respectively, colored by charge on the solvent exposed surfaces as calculated with the Adaptive Poisson-Boltzmann Solver (APBS) [38, 39].

It has been suggested that BlsA and related short BLUF proteins may transduce the light signal by the interaction of the FAD adenine ring with a cognate effector, as proposed for the BlrB protein [42], or the interaction of the CT regions of BLUF photoreceptors with putative protein partners involved in downstream signal transduction pathways that result in differential gene expression in response to illumination. The latter possibility is supported by the apparent effect that replacing the K144 or K145 charged residues has on light-regulated surface motility without significantly affecting flavin binding and photocycling. This outcome could be explained by localized changes in the electrostatic properties of BlsA’s surface. The K144E and K145E mutations result in significant perturbations to the electrostatic surface near the predicted ⍺_5_ helix (Fig 6).

This surface area is strongly positively charged for wild type BlsA. However introduction of either the K144E or K145E mutations substantially alters surface-accessible charges, which results in a surface with an additional negatively charged character due to the surface side chain carboxylates of K144E and K145E. Based on these observations, we speculate that these changes in charge for the solvent-exposed surface alter the interaction of BlsA with downstream partners responsible for regulation of gene expression in response to illumination. This hypothesis could be supported by the recent observation indicating that BlsA interacts with the Fur iron-dependent regulator to control the transcriptional expression of *A*. *baumannii* genes coding for acinetobactin-mediated iron acquisition functions in a temperature-dependent manner [51]. Confirming the proposed BlsA-Fur interactions and testing the iron response of the OR.K144E and OR.K145E mutants under conditions similar to those described by Tuttobene *et al*. [51] should provide critical information needed to understand these interactions and shed some light on the mechanism(s) BlsA uses to regulate a wide range of cellular functions in response to illumination.

## Supporting information

S1 Fig Predicted secondary structures of short BLUF proteins. The secondary structures of BlsA, SnfB, BlrB, T110078 and Slr1694 were predicted using the SABLE server. Green arrows and red wavy lines represent β-strands and α-helices, respectively.
(TIF)

S2 Fig Growth curves of the 17978 parental strain and isogenic complemented derivatives. Cells of the 17978 strain (17978) and the OR derivative expressing BlsA with either the Q51A or W92A mutations, were cultured in SA at 24°C under darkness in a shaking incubator. The OD_600_ of each culture was determined hourly for 12 h and then at 24 h after inoculation using two independent cultures of each tested strain. Error bars represent the standard error of each data set.
(TIF)

S3 Fig Overexpression of His-tagged BlsA and derivatives in *E*. *coli* BL21. SDS-PAGE and immunoblot analysis of whole cell lysates of uninduced cells, lane 2, and induced cells harboring pMU1254 (parental BlsA), lane 3; pMU1273 (Y7A), lane 4; pMU1255 (Q51A), lane 5; pMU1271 (Y7A/Q51A), lane 6; or pMU1298 (W92A), lane 7. The BlsA amino acid changes coded for by each pET-15b derivative is indicated in parentheses. Lane 1, molecular weight markers. Total proteins were detected by Coomassie Blue staining (A) and BlsA was detected by immunoblotting with anti-BlsA polyclonal antibodies (B).
(TIF)

S4 Fig BlsA responses to low and high light intensities. Difference of light minus dark spectra of purified WT BlsA protein samples illuminated at three different light intensities. Red line, 20 μmol/m^2^/s; blue line, 100 μmol/m^2^/s, black line, 200 μmol/m^2^/s.
(TIF)

S5 Fig CD spectra of dBlsA, lBlsA and related derivatives. Purified 3 μM samples of each protein dissolved in 10 mM phosphate buffer, pH 8.0 containing 20 mM NaCl were analyzed as described in Materials and methods.
(TIF)

S6 Fig HPLC of flavin standards. A volume of 10 μl of 100 μM stock solutions of FAD, FMN and Ribo was injected into a Waters Symmetry C18 reversed-phase column and eluted and detected as described in Materials and methods. Retention times (min) for each standard is indicated in the cognate panels.
(TIF)

S7 Fig Detection of flavins bound to BlsA. HPLC of heat-denatured supernatants of purified His-tagged BlsA recombinant derivatives generated by site-directed deletions (BlsA.Δ152–156 and BlsA.Δ143–156) or point mutations (BlsA.K144E and BlsA.K145E). The retention times for each flavin component is indicated in minutes.
(TIF)

S1 Table Primers used in this study. (DOCX)
