# Supplementary figures and images for: Structural and functional analysis of the Acinetobacter baumannii BlsA photoreceptor and regulatory protein
Source: PLoS One. 2019 Aug 15;14(8):e0220918. doi: 10.1371/journal.pone.0220918 (PMC6695109; doi:10.1371/journal.pone.0220918)

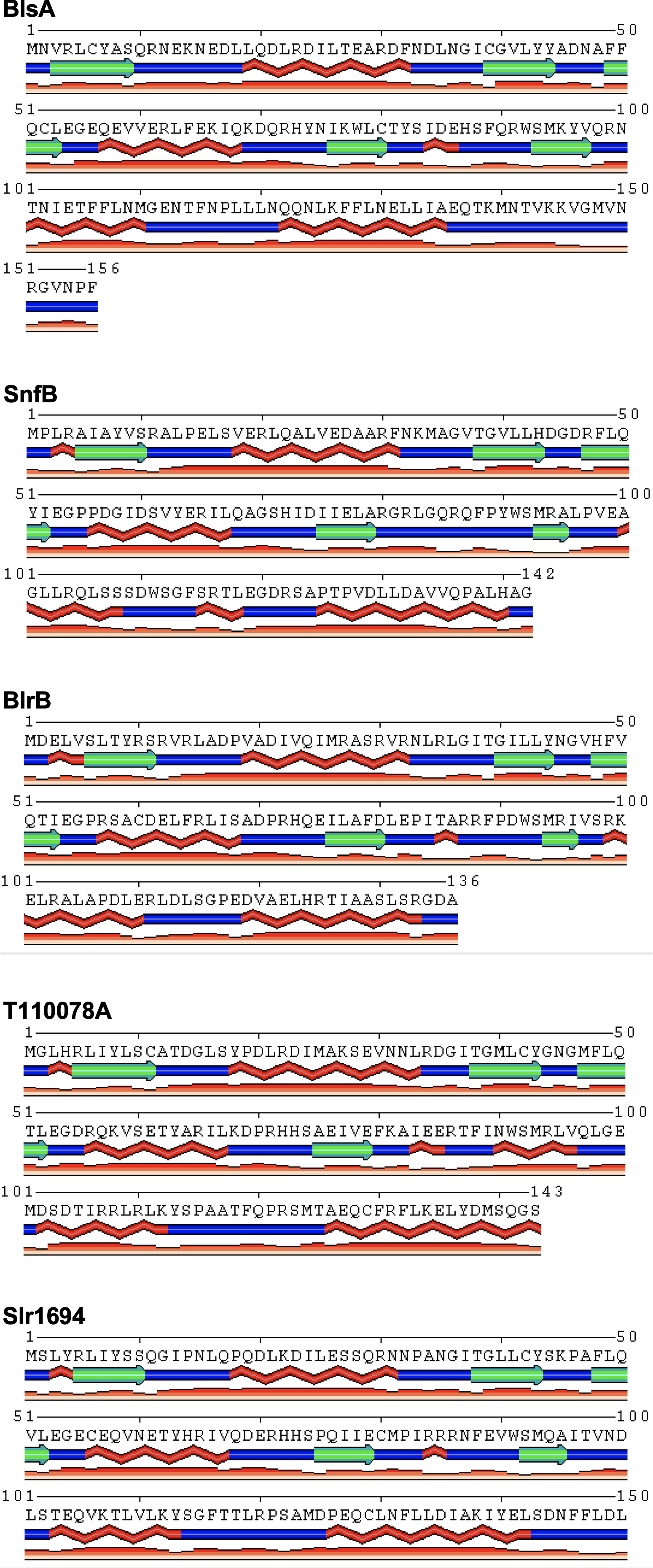

Supplement: S1 Fig — The secondary structures of BlsA, SnfB, BlrB, T110078 and Slr1694 were predicted using the SABLE server. Green arrows and red wavy lines represent β-strands and α-helices, respectively. (TIF) [file pone.0220918.s001.tif]

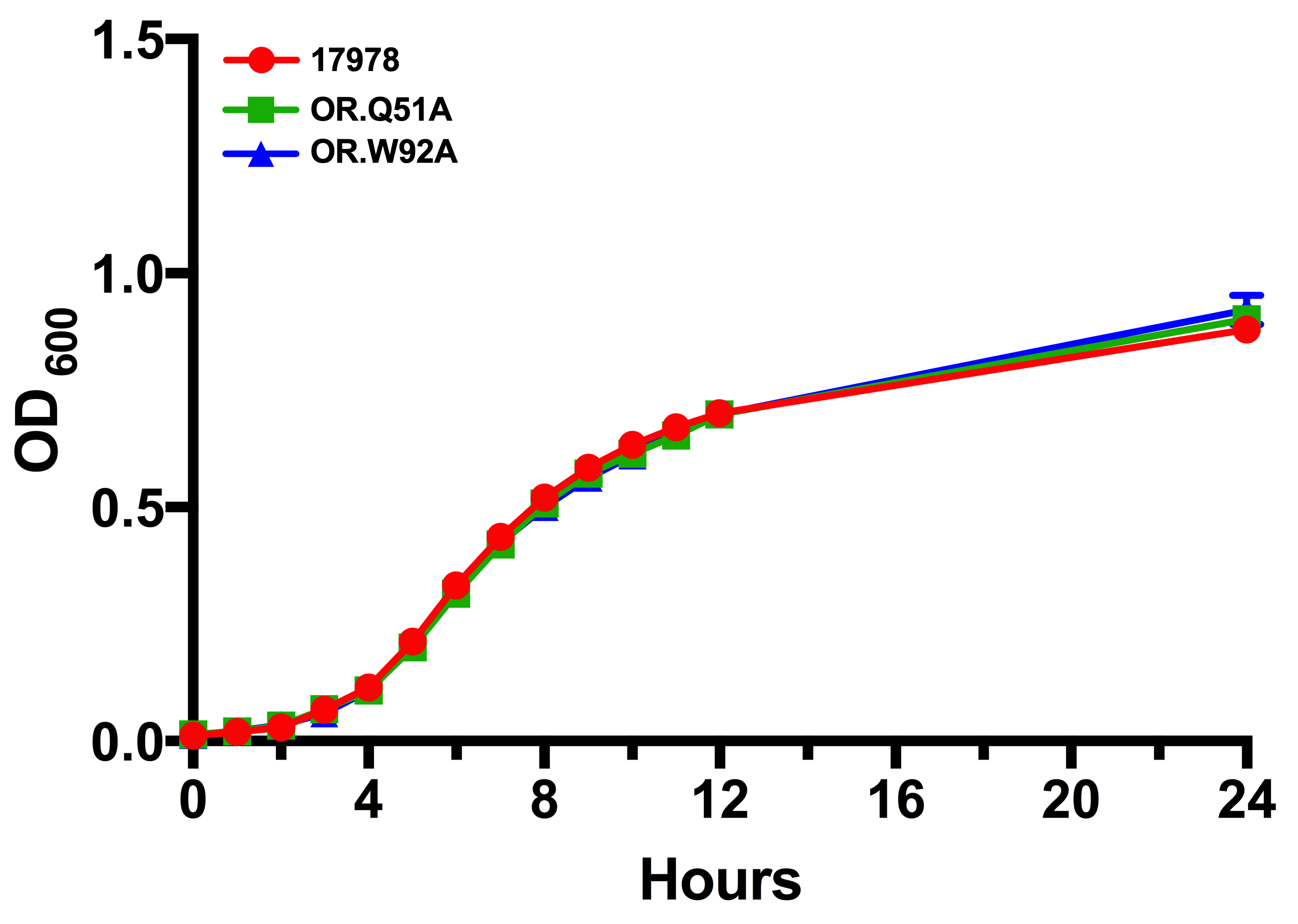

Supplement: S2 Fig — Cells of the 17978 strain (17978) and the OR derivative expressing BlsA with either the Q51A or W92A mutations, were cultured in SA at 24°C under darkness in a shaking incubator. The OD600 of each culture was determined hourly for 12 h and then at 24 h after inoculation using two independent cultures of each tested strain. Error bars represent the standard error of each data set. (TIF) [file pone.0220918.s002.tif]

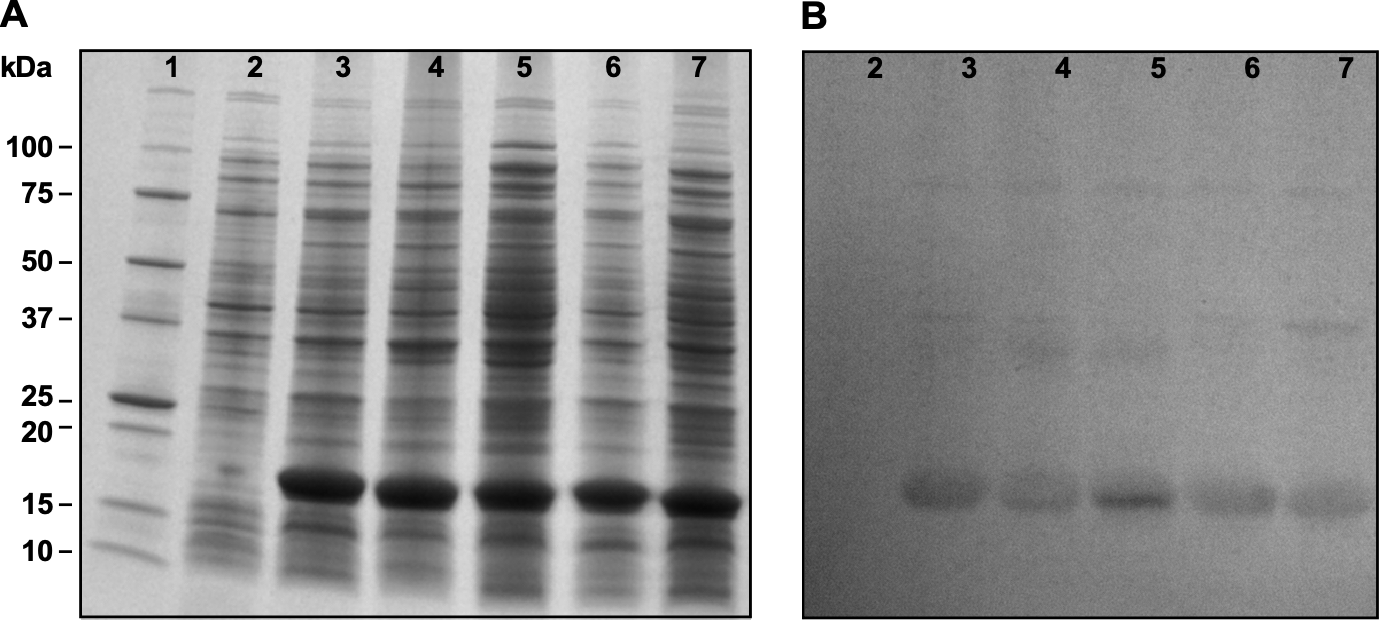

Supplement: S3 Fig — SDS-PAGE and immunoblot analysis of whole cell lysates of uninduced cells, lane 2, and induced cells harboring pMU1254 (parental BlsA), lane 3; pMU1273 (Y7A), lane 4; pMU1255 (Q51A), lane 5; pMU1271 (Y7A/Q51A), lane 6; or pMU1298 (W92A), lane 7. The BlsA amino acid changes coded for by each pET-15b derivative is indicated in parentheses. Lane 1, molecular weight markers. Total proteins were detected by Coomassie Blue staining (A) and BlsA was detected by immunoblotting with anti-BlsA polyclonal antibodies (B). (TIF) [file pone.0220918.s003.tif]

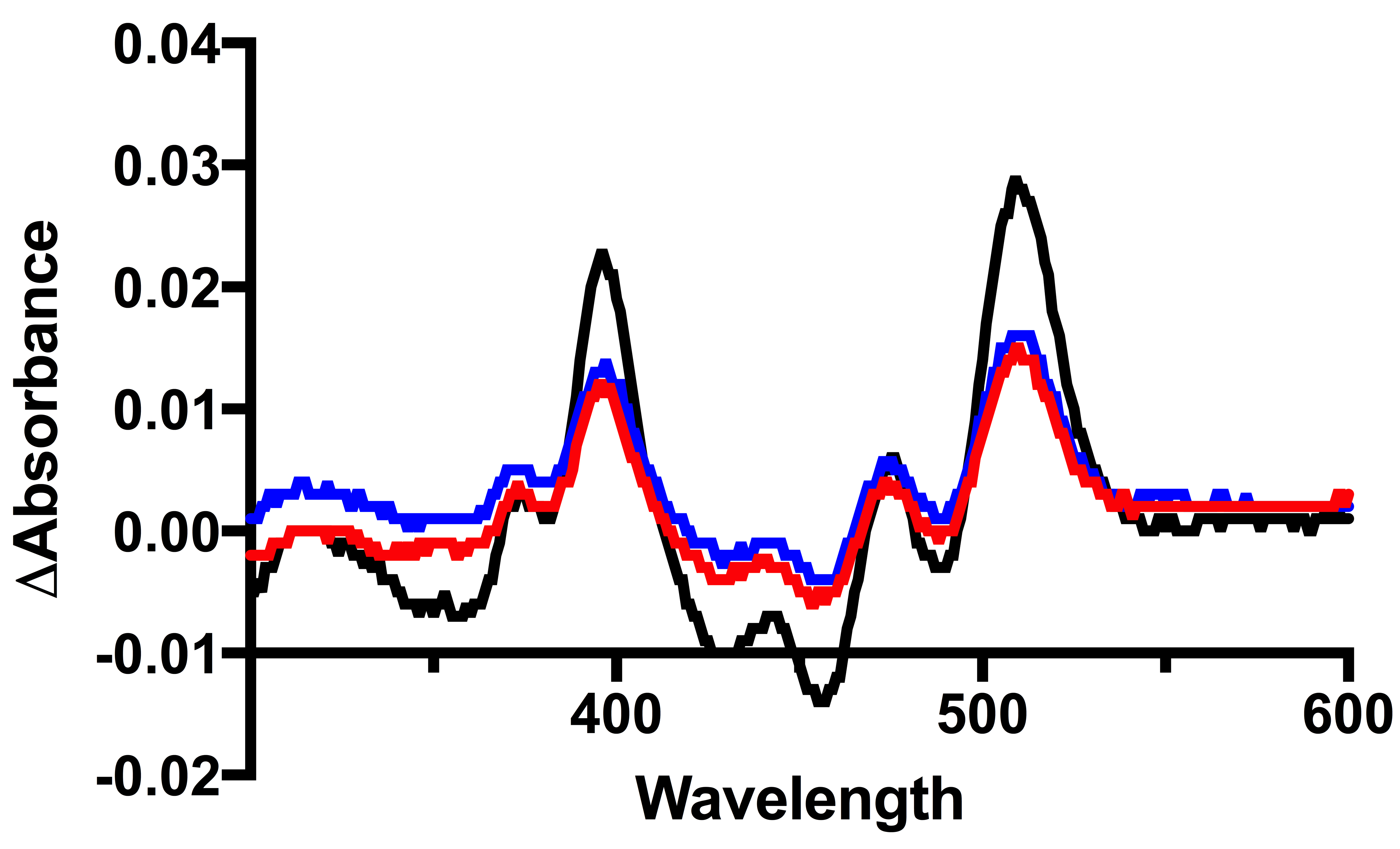

Supplement: S4 Fig — Difference of light minus dark spectra of purified WT BlsA protein samples illuminated at three different light intensities. Red line, 20 μmol/m2/s; blue line, 100 μmol/m2/s, black line, 200 μmol/m2/s. (TIF) [file pone.0220918.s004.tif]

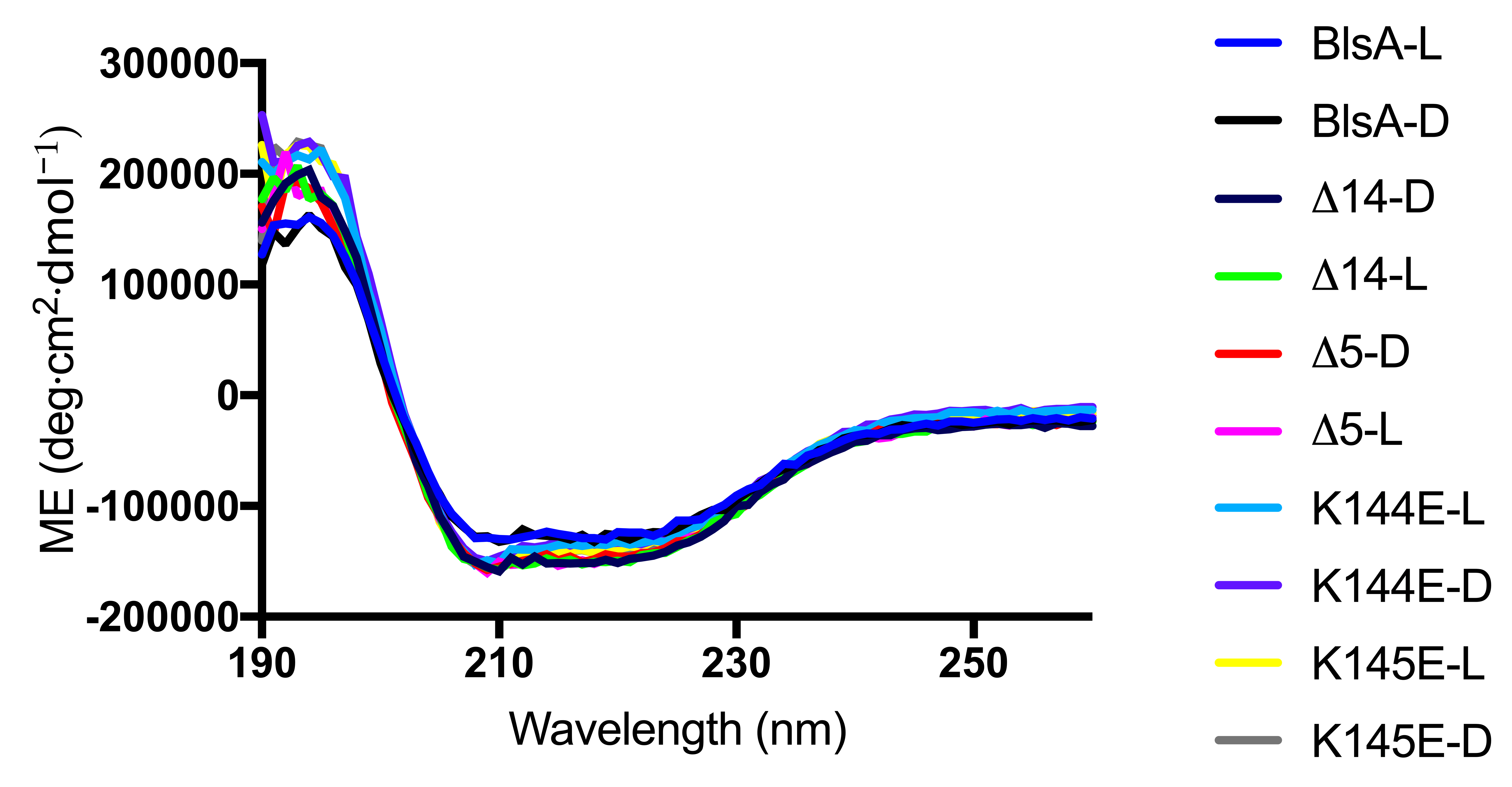

Supplement: S5 Fig — Purified 3 μM samples of each protein dissolved in 10 mM phosphate buffer, pH 8.0 containing 20 mM NaCl were analyzed as described in Materials and methods. (TIF) [file pone.0220918.s005.tif]

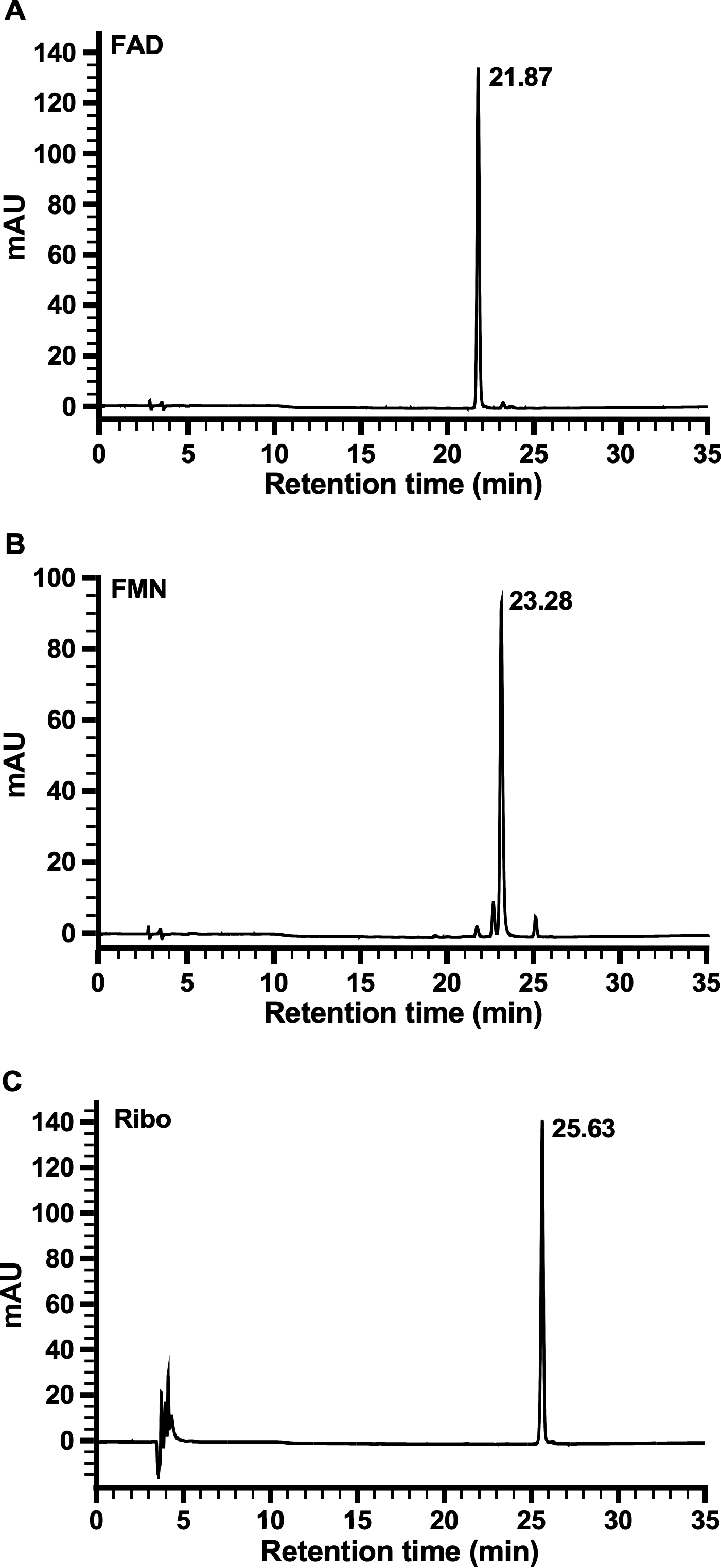

Supplement: S6 Fig — A volume of 10 μl of 100 μM stock solutions of FAD, FMN and Ribo was injected into a Waters Symmetry C18 reversed-phase column and eluted and detected as described in Materials and methods. Retention times (min) for each standard is indicated in the cognate panels. (TIF) [file pone.0220918.s006.tif]

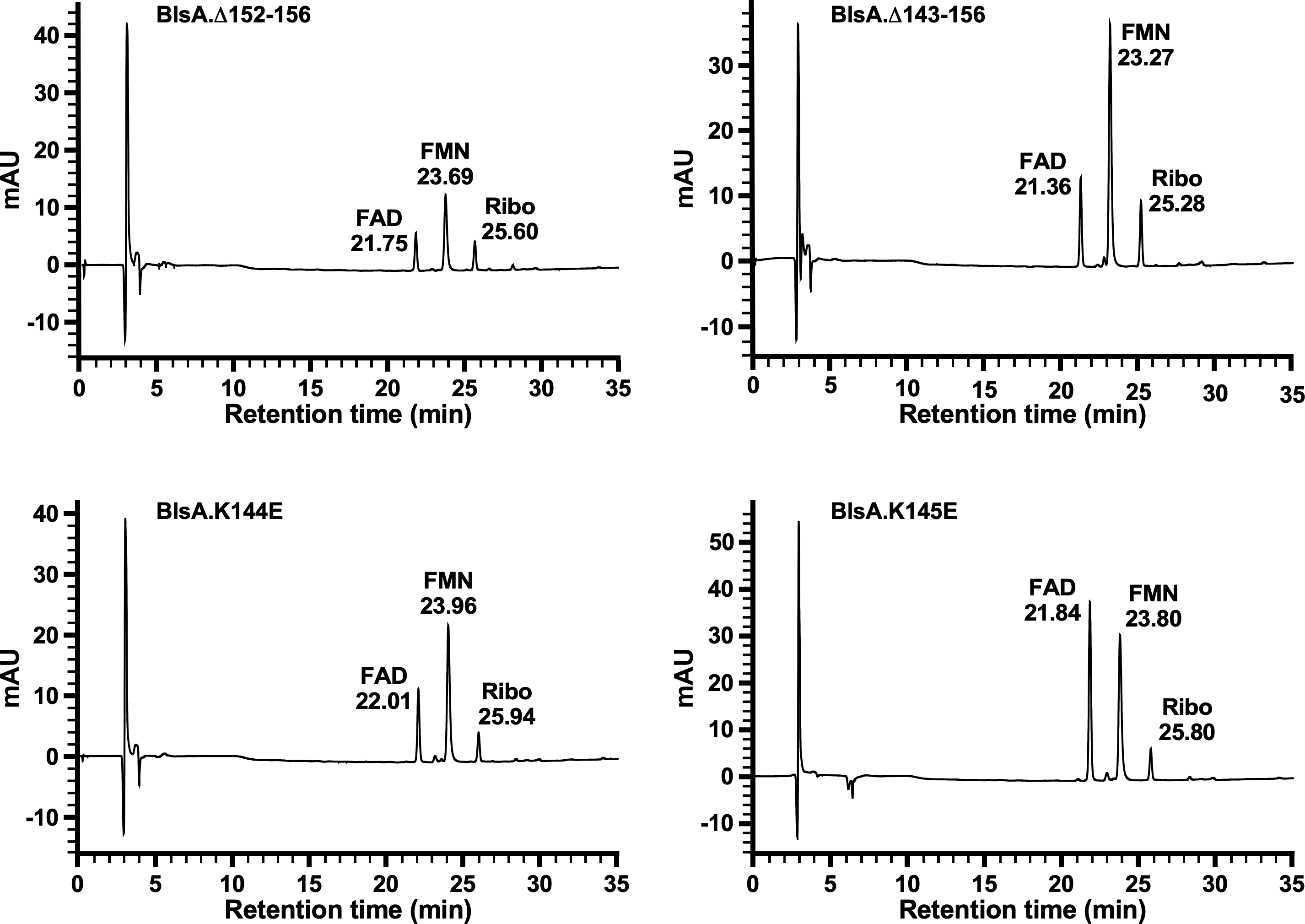

Supplement: S7 Fig — HPLC of heat-denatured supernatants of purified His-tagged BlsA recombinant derivatives generated by site-directed deletions (BlsA.Δ152–156 and BlsA.Δ143–156) or point mutations (BlsA.K144E and BlsA.K145E). The retention times for each flavin component is indicated in minutes. (TIF) [file pone.0220918.s007.tif]
