# Supplementary material for: Structural and functional analysis of the Acinetobacter baumannii BlsA photoreceptor and regulatory protein
Source: PLoS One. 2019 Aug 15;14(8):e0220918. doi: 10.1371/journal.pone.0220918 (PMC6695109; doi:10.1371/journal.pone.0220918)
Supplement: S1 Table — (DOCX) [file pone.0220918.s008.docx]

**S1 Table. Primers used in this study.**

| Primer number | Sequence^a^ |
| --- | --- |
| 4379 | 5’-ATGTGAGGATCCAGTATTACAAATTGAACGTG-3’ |
| 4380 | 5’-TGATATGGATCCGTGATATGGATGCTGTCG-3’ |
| 4383 | 5’-CCGATTCACCATTCCAAC-3’ |
| 4384 | 5’-CGTATTCATCTTAGTTTGCTC-3’ |
| 4385 | 5’-TAGCTATTAGATGTGATACCG-3’ |
| 4386 | 5’-CAGCAAAAGCGGGTTAAAG-3’ |
| 4387 | 5’-GAGCAAACTAAGATGAATACGG-3’ |
| 4388 | 5’-CGTTTAAATCGTTGAAATCACGACTTTCTGTCAGAATATCACGTAAATCTT-3’ |
| 4392 | 5’-CACAAATCCCGTTTAAATCGTTGTTATCACGAGCTTCTGTCVAGAATAT-3’ |
| 4394 | 5’-GTTCACCTTCTAGACATGCAAAAAATGCATTATCGGCATAATAAAGCACCC-3’ |
| 4396 | 5’-CGTTGGCTGGCAGCACACAGGCGAACGTTCATAAGAC-3’ |
| 4412 | 5’-GATCAAAGGCATTATAATATTAAAGCGTTATGCACATACTCCATTGAT-3’ |
| 4421 | 5’-AATTAAGAGTTCATTTAAG-3’ |
| 4423 | 5’-ATGGTGAATCGGGGA-3’ |
| 4425 | 5’-CAAACTAAGATGAATACGGTTAAAGAAGTTGGAATGGTGAATCGGG-3’ |
| 4426 | 5’-ATGAACACTCTTTTCAGCGTGCGTCAATGAAATACGTGCAGC-3’ |
| 4427 | 5’-TAGTGACATATGAACGTTCGCCTGTGT-3’ |
| 4428 | 5’-TGATATGGATCCCTAGAACGGGTTTACTCC-3’ |
| 4439 | 5’-TAGGGATCCGGCTGCTAAC-3’ |
| 4440 | 5’-CGTATTCATCTTAGTTTGCTCTGC-3’ |
| 4441 | 5’-CCGATTCACCATTCCAACTT-3’ |
| 4478 | 5’-GCAAACTAAGATGAATACGGTTGAAAAAGTTGGAATGGTGAATCG-3’ |

^a^Underlined nucleotides identify *Bam*HI and *Nde*I restriction sites.
